# Seroprevalence of Lyme Borreliosis in Europe: Results from a Systematic Literature Review (2005–2020)

**DOI:** 10.1089/vbz.2022.0069

**Published:** 2023-04-12

**Authors:** Leah Burn, Andreas Pilz, Andrew Vyse, Aura Victoria Gutiérrez Rabá, Frederick J. Angulo, Thao Mai Phuong Tran, Mark A. Fletcher, Bradford D. Gessner, Jennifer C. Moïsi, James H. Stark

**Affiliations:** ^1^ P95 Pharmacovigilance & Epidemiology, Princeton, New Jersey, USA.; ^2^ Pfizer Global Medical Affairs, Vaccines, Vienna, Austria.; ^3^ Pfizer Vaccines Medical, Walton Oaks, United Kingdom.; ^4^ P95 Pharmacovigilance & Epidemiology, Leuven, Belgium.; ^5^ Pfizer Vaccines Medical Development, Scientific and Clinical Affairs, Collegeville, Pennsylvania, USA.; ^6^ Pfizer Emerging Markets Medical Affairs, Vaccines, Paris, France.; ^7^ Pfizer Medical Development, Scientific and Clinical Affairs, Vaccines, Paris, France.

**Keywords:** Lyme borreliosis, seroprevalence, seropositivity, Europe, epidemiology, diagnostics

## Abstract

**Background:**

Lyme borreliosis (LB), a tick bite-transmitted infection caused by *Borrelia burgdorferi sensu lato* (*Bbsl*) complex spirochetes, is the most common tickborne disease in Europe. Studies in European countries have reported LB seroprevalence (prevalence of antibodies to *Bbsl* infection) and diagnostic strategies used for testing. We conducted a systematic literature review to summarize contemporary LB seroprevalence in Europe.

**Methods:**

PubMed, Embase, and CABI Direct (Global Health) databases were searched from 2005 to 2020 to identify studies reporting LB seroprevalence in European countries. Reported single-tier and two-tier test results were summarized; algorithms (standard or modified) were used to interpret final test results from studies that used two-tier testing.

**Results:**

The search yielded 61 articles from 22 European countries. Studies used a range of diagnostic testing methods and strategies (48% single-tier, 46% standard two-tier, and 6% modified two-tier). In 39 population-based studies, of which 14 were nationally representative, seroprevalence estimates ranged from 2.7% (Norway) to 20% (Finland). There was substantial heterogeneity among studies in terms of design, cohort types, periods sampled, sample sizes, and diagnostics, which limited cross-study comparisons. Nevertheless, among studies that reported seroprevalence in persons with greater exposure to ticks, LB seroprevalence was higher among these groups than in the general population (40.6% vs. 3.9%). Furthermore, among studies that used two-tier testing, general population LB seroprevalence was higher in Western Europe (13.6%) and Eastern Europe (11.1%) than in Northern Europe (4.2%) and Southern Europe (3.9%).

**Conclusion:**

Despite variations in LB seroprevalence between and within European subregions and countries, high seroprevalence was observed in certain geographic regions and particular risk groups, suggesting significant disease burden and supporting the need for improved, targeted public health interventions such as vaccination. Harmonized approaches to serologic testing and more nationally representative seroprevalence studies are needed to better understand the prevalence of *Bbsl* infection in Europe.

## Introduction

Lyme borreliosis (LB), the most common tickborne disease in Europe, is caused by an infection with spirochetes of the *Borrelia burgdorferi sensu lato* (*Bbsl*) complex, which is transmitted to humans through the bite of a vector-competent, infected tick (Bennet et al. 2007, Bobe et al. 2021). Although a proportion of infections are reported to be asymptomatic (median proportion of asymptomatic infected persons from studies in Europe was 50%) (Stanek et al. 2011, Hofhuis et al. 2013, Markowicz et al. 2021), asymptomatic persons can still seroconvert (Huegli et al. 2011, Hofhuis et al. 2013, Wilhelmsson et al. 2016). Clinical manifestations of LB typically begin with development of erythema migrans at the site of a tick bite, with signs and symptoms such as fatigue, fever, arthralgia, and myalgia (Bobe et al. 2021). The infection may then disseminate and present with a range of clinical syndromes, including neurologic (Lyme neuroborreliosis), rheumatologic (Lyme arthritis), dermatologic (acrodermatitis chronica atrophicans), hematologic (borrelial lymphocytoma), and/or cardiac (Lyme carditis) (Steere et al. 2016).

The distribution and density of vector-competent ticks infected with clinically relevant *Bbsl* in Europe has been widely investigated. Vector-competent ticks mainly include *Ixodes ricinus* and *Ixodes persulcatus* (Margos et al. 2019, Perry 2021), and eight clinically relevant genospecies belonging to *Bbsl* complex have been reported: *B. burgdorferi sensu stricto*, *Borrelia afzelii*, *Borrelia garinii*, *Borrelia valaisiana*, *Borrelia lusitaniae*, *Borrelia bissettii*, *Borrelia bavariensis*, and *Borrelia spielmanii* (Rauter and Hartung 2005, Richter and Matuschka 2006, Margos et al. 2009). Living or working in highly endemic geographic areas and undertaking outdoor occupations or leisure activities (*e.g.,* forestry work, farming, hunting, hiking) lead to greater risk of exposure (Piacentino and Schwartz 2002, Magnavita et al. 2022).

**FIG. 1.**
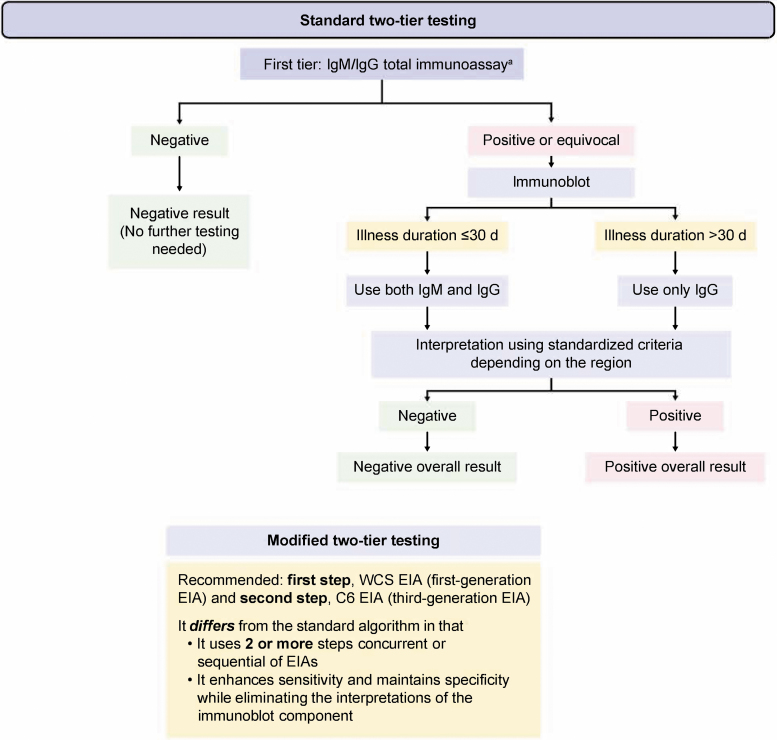
Algorithm for the serologic testing of LB (Centers for Disease Control and Prevention 2011, Marques 2018, Branda and Steere 2021). Overall test results should be used from two-tier testing, with the original denominator. ^a^EIA or IFA WCS. C6, C6 protein of the variable major protein-like sequence lipoprotein; EIA, enzyme immunoassay; IFA, immunofluorescence assay; IgG, immunoglobulin G; IgM, immunoglobulin M; LB, Lyme borreliosis; WCS, whole-cell sonicate.

The serologic response to *Bbsl* infection begins with immunoglobulin M (IgM) antibodies, which initially become detectable within a few days to a few weeks after infection (Steere et al. 2016). Immunoglobulin class switching occurs, and immunoglobulin G (IgG) antibodies become detectable within 1–2 months; serum IgG and IgM antibodies can still be detected 10–20 years after infection (Kalish et al. 2001, Peltomaa et al. 2003, Glatz et al. 2006). Most individuals infected by *Bbsl* develop detectable antibodies; therefore, serology is the standard laboratory method for confirming an LB diagnosis (Wilske et al. 2007, Steere et al. 2016). Serologic confirmation of an LB diagnosis uses a two-tier strategy to optimize sensitivity and specificity (Fig. 1) (Branda and Steere 2021). For standard two-tier testing, the first-tier screen for IgG or IgM is an enzyme immunoassay (EIA; or enzyme-linked immunoassay [ELISA]) or an immunofluorescence assay (IFA).

To maximize specificity in the second tier, first-tier positive samples are then tested by western blot (WB) for IgM and/or IgG (Branda and Steere 2021). EIAs that detect antibodies to the highly conserved variable major protein-like sequence expressed (VlsE) peptides can improve specificity (Branda et al. 2013). Alternatively, modified two-tier testing methods that use two or more different EIA or ELISA methods as the second tier can improve diagnostic sensitivity without sacrificing specificity (Branda and Steere 2021).

Population-level seroprevalence can be used to monitor the prevalence of current or past infection in European countries without public health surveillance for LB. Furthermore, seroprevalence estimates, which indicate exposure to *Bbsl* at some point, can be compared with the number of surveillance-reported symptomatic LB cases to assess under-ascertainment of human contact with vector-competent, *Bbsl*-infected ticks. Seroprevalence data are capable of identifying geographic localities and outdoor occupations or leisure activities at higher risk of infection and monitoring population-level changes over time, such as those related to public health interventions. Consequently, we conducted a systematic literature review to obtain contemporary estimates of the seroprevalence of LB in Europe.

## Methods

The methodology, search strategy, and inclusion and exclusion criteria for the systematic literature review and analysis are described in detail in a protocol developed by the Pfizer/P95 Lyme Review Group according to Preferred Reporting Items for Systematic Reviews and Meta-Analyses (PRISMA, 2020) guidelines and registered in PROSPERO (CRD42021236906).

### Search strategy

We conducted a multidatabase, systematic literature review across PubMed, Embase, and CABI Direct (Global Health) databases from January 1, 2005, to November 20, 2020, using the following search terms (with no language restrictions): Lyme, *Borrelia*, and borreliosis. After duplicates were removed, titles and abstracts were screened independently by two reviewers for their relevance to the study objectives, and the selected full-text articles were assessed based on predefined inclusion and exclusion criteria by two reviewers. Full-text articles published in other European languages were translated into English using DeepL (DeepL SE 2021).

### Inclusion and exclusion criteria

Articles reporting results of serologic testing for *Bbsl* complex infection were included if the articles clearly reported a defined numerator (number of seropositive cases), denominator (size of the population tested), and diagnostic testing strategy based on at least one diagnostic test of either an EIA or ELISA, IFA, or western immunoblot.

Health-economic and cost studies, studies of biomedical mechanisms, mathematical models, and diagnostic guidelines, and studies without human serologic results were excluded, as were data only in abstract form from conferences, letters, perspective or opinion articles, or commentaries. Literature review articles were not included but were scanned for additional references.

Articles retrieved using the above criteria were further assessed to select articles reporting seroprevalence estimates. Studies were included if they reported estimates of seroprevalence (and not disease incidence) based on rigorous population sampling methods such as random, cross-sectional, or survey cluster sampling. If multiple seroprevalence results were obtained over time, only the first seroprevalence estimate was used to avoid mixing seroprevalence with seroconversion or incidence. Studies were excluded if they comprised participants who were selected for participation because they had suspected or confirmed LB or if they reported data from cohorts recruited based on tick bite exposure.

### Data extraction

Relevant variables from selected articles were extracted into DistillerSR (Evidence Partners 2021). A reviewer independently confirmed the extraction by reviewing 20% of the articles. Predefined outcomes of interest for extraction were testing method, diagnostic strategy, results of serologic testing, population characteristics, and risk status. All discrepancies were discussed and resolved by the authors. The outcome of interest was seropositivity by at least one serologic test. Population risk level was defined by likelihood of exposure to ticks due to residence in specific regions or by occupational or leisure activities.

### Data interpretation and analysis

Study results were synthesized by relevant descriptive variables and seroprevalence results. When studies used several types of single-tier tests (*e.g.,* measurement of IgM or IgG), IgG tests results were preferred. This is because IgM-based tests, although useful for clinical diagnosis of early infection, are more likely to yield false positives (Branda and Steere 2021). In this review, therefore, IgG results were used to report seroprevalence. When studies used a two-tier testing strategy, final test results were based on calculations from standard or modified algorithms (Fig. 1). More prominence was given to test results from two-tier strategies due to the optimal sensitivity and specificity of this system (Branda and Steere 2021).

The 95% confidence intervals (CIs) (Cisak et al. 2008) for seroprevalence estimates were calculated using the binomial exact CI for a single proportion. Seroprevalence results were stratified by age, sex, and intracountry region (Supplementary Table S1).

We calculated the population-weighted mean seroprevalence for each of the European regions using data available for countries belonging to that region. If a study reported data on both the national and regional levels, we used only the data on the national level as a summary of that country. For studies that reported regional, subnational data only, seroprevalence from the largest reported region was used to calculate the weighted mean seroprevalence. We stratified weighted mean seroprevalence by diagnostic strategy and antibody testing method (Tables 5–8). Results were also stratified by exposure risk group. A population was considered a high-risk group for LB if it included a group with high exposure to ticks, such as forestry workers, farmers, or residents in high-risk regions (Tables 1–4).

**Table 1.** Estimates of Lyme Borreliosis Seroprevalence in Eastern Europe from Published Literature, 2005–2020

Country	National or region within country	References	Study design (data collection period)	Sampling method	Sample size, N	Cohort description	Age group, years	Diagnostic testing strategy	Type of diagnostic test	Final SP result, %^a^
Czech Republic	Plzen, Vysocina, South Bohemia, Central Bohemia, South Moravia, Moravia-Silesia	Kříž et al. (2018)	Cross-sectional (1978–1989) Cross-sectional (2001)	Random	434	Serum bank samples^b^	Not described	Single tier	ELISA IgG	25.1
					270	Serum bank samples^b^		Single tier	ELISA IgG	10.3
Czech Republic	Prague	Hajek et al. (2006)	Cross-sectional (1995–1999)	Consecutive	890	Patients with psychiatric disorders^b^	Mean (SD): 33.6 (13.9)	Single tier	ELISA IgM and/or IgG	35.7
Czech Republic	Prague	Kuchynka et al. (2016)	Cross-sectional (2013–2014)	Convenience	50	Adults with normal left ventricular systolic function and no history suggestive of myocarditis^b^	Mean (SD): 67 (9)	Standard two-tier	ELISA IgG and/or IgM+WB	14
Hungary	National^c^	Lakos et al. (2012)	Cross-sectional (1992–1995)	Convenience	1670	Forestry workers^d^	Mean (SD): 40.7 (10.2)	Single tier	ELISA IgG+IgM	37.2
Poland	Southern Podlasie Lowland, Lublin Polesie	Tokarska-Rodak et al. (2014)	Cross-sectional (2013)	Not described	172	Forestry workers^d^	Range: 25–72	Standard two-tier	ELISA+WB IgG and/or IgM	54.9
					104	Farmers^d^		Standard two-tier	ELISA+WB IgG and/or IgM	28.0
					45	Persons not occupationally exposed to ticks^b^		Standard two-tier	ELISA+WB IgG and/or IgM	5.0
Poland	Warsaw	Machcińska et al. (2013)	Cross-sectional (2013)	Not described	90	Blood donors^b^	Range: 18–70	Single tier	ELISA IgM	2.2
									ELISA IgG	10.0
Poland	Six forest districts in Ostróda	Kocbach and Kocbach (2014)	Cross-sectional (2011–2012)	Convenience	332	Forestry workers^d^	Mean (range): 37 (25–67)	Standard two-tier	ELISA+WB IgG	27.4
	St. Jablonki				42			Standard two-tier	ELISA+WB IgG	11.9
	Kudypy				47			Standard two-tier	ELISA+WB IgG	25.5
	Wipsowo				79			Standard two-tier	ELISA+WB IgG	22.8
	Wichrowo				54			Standard two-tier	ELISA+WB IgG	31.5
	Iława				47			Standard two-tier	ELISA+WB IgG	21.3
	Miłomłyn				63			Standard two-tier	ELISA+WB IgG	23.8
Poland	Western Poland (Międzychód)	Bura et al. (2018)	Cross-sectional (2014)	Not described	48	Forest rangers^d^	Mean (SD): 45.0 (9.6)	Standard two-tier	ELISA+WB IgM	8.3
								Standard two-tier	ELISA+WB IgG	37.5
Poland	National^c^	Pawelczyk et al. (2019)	Cross-sectional (2013/2016)	Not described	227	HIV-infected persons^b^	Mean (range): 33 (20–51)	Single tier	ELISA IgM	29.1
									ELISA IgG	4.8
					199	Healthy blood donors^b^	Mean (range): 36 (18–71)	Single tier	ELISA IgM	13.1
									ELISA IgG	5.0
Poland	Northeastern (Białowieza Primeval forest), Southern (Radomsko forest)	Podsiadly et al. (2011)	Prospective cohort (2008–2009)	Not described	129	Forestry workers^d^	Not described	Standard two-tier	ELISA+WB IgG	34.0
Poland	Lublin	Cisak et al. (2008)	Cross-sectional (1998–2007)	Not described	94	Farmers^d^	Mean (SD): 56.3 (14.3)	Standard two-tier	ELISA+WB IgG and/or IgM	32.9
					50	Blood donors^b^	Mean (SD): 29.7 (5.0)	Standard two-tier	ELISA+WB IgG and/or IgM	6.0
Poland	National^c^	Zając et al. (2017)	Cross-sectional (2015–2016)	Convenience	3597	Farmers^d^	Mean (SD): 51.3 (11.4)	Single tier	ELISA IgM	11.5
									ELISA IgG	13.7
	Gostynin				150			Single tier	ELISA IgM	13.3
	Gostynin				150			Single tier	ELISA IgG	14.7
	Siedlce				329			Single tier	ELISA IgM	10.3
	Siedlce				329			Single tier	ELISA IgG	16.7
	Kolno				120			Single tier	ELISA IgG	14.2
	Kolno				120			Single tier	ELISA IgM	6.7
	Siemiatycze				106			Single tier	ELISA IgM	10.4
	Siemiatycze				106			Single tier	ELISA IgG	19.8
	Biała Podlaska				120			Single tier	ELISA IgM	14.2
	Biłgoraj				59			Single tier	ELISA IgM	11.9
	Chełm				120			Single tier	ELISA IgM	8.3
	Kraśnik				317			Single tier	ELISA IgM	8.5
	Puławy				103			Single tier	ELISA IgM	9.7
	Radzyń Podlaski				114			Single tier	ELISA IgM	19.3
	Włodawa				150			Single tier	ELISA IgM	25.3
	Zamość				99			Single tier	ELISA IgM	8.1
	Węgrów				182			Single tier	ELISA IgM	8.8
	Hajnówka				103			Single tier	ELISA IgM	20.4
	Augustów				150			Single tier	ELISA IgM	6.0
	Mońki				99			Single tier	ELISA IgM	7.1
	Suwałki				135			Single tier	ELISA IgM	12.6
	Biała Podlaska				120			Single tier	ELISA IgG	20.0
	Biłgoraj				59			Single tier	ELISA IgG	10.2
	Chełm				120			Single tier	ELISA IgG	5.8
	Kraśnik				317			Single tier	ELISA IgG	11.4
	Puławy				103			Single tier	ELISA IgG	14.0
	Radzyń Podlaski				114			Single tier	ELISA IgG	16.7
	Włodawa				150			Single tier	ELISA IgG	24.0
	Zamość				99			Single tier	ELISA IgG	16.2
	Węgrów				182			Single tier	ELISA IgG	13.7
	Hajnówka				103			Single tier	ELISA IgG	20.4
	Augustów				150			Single tier	ELISA IgG	12.7
	Mońki				99			Single tier	ELISA IgG	10.2
	Suwałki				135			Single tier	ELISA IgG	18.5
Poland	Southern Poland	Buczek et al. (2009)	Cross-sectional (2003–2006)	Convenience	291	Office workers^b^	Not described	Single tier	ELISA IgM	10.0
									ELISA IgG	13.7
					864	Forestry workers^d^			ELISA IgM	13.8
									ELISA IgG	25.0
	Gidle				216			Single tier	ELISA IgM	6.0
	Herby				203			Single tier	ELISA IgM	4.9
	Kłobuck				160			Single tier	ELISA IgM	1.25
	Koniecpol				211			Single tier	ELISA IgM	6.2
	Przedbórz				141			Single tier	ELISA IgM	6.4
	Złoty Potok				224			Single tier	ELISA IgM	17.4
	Gidle				216			Single tier	ELISA IgG	21.3
	Herby				203			Single tier	ELISA IgG	22.2
	Kłobuck				160			Single tier	ELISA IgG	20.6
	Koniecpol				211			Single tier	ELISA IgG	23.7
	Przedbórz				141			Single tier	ELISA IgG	14.2
	Złoty Potok				224			Single tier	ELISA IgG	14.3
Poland	Eleven forest inspectorates	Kiewra et al. (2018)	Cross-sectional (2016)	Not described	646	Forestry workers^d^	Range: 21–67	Standard two-tier	ELISA+WB IgM	8.6
								Standard two-tier	ELISA+WB IgG	17.8
	Bardo				49			Standard two-tier	ELISA+WB IgM	8.1
	Legnica				51			Standard two-tier	ELISA+WB IgM	15.6
	Milicz				89			Standard two-tier	ELISA+WB IgM	6.7
	Henryków				34			Standard two-tier	ELISA+WB IgM	2.9
	Jugów				59			Standard two-tier	ELISA+WB IgM	8.4
	Oleśnica				96			Standard two-tier	ELISA+WB IgM	12.5
	Ruszów				65			Standard two-tier	ELISA+WB IgM	6.1
	Śnieżka				48			Standard two-tier	ELISA+WB IgM	8.3
	Świeradów				55			Standard two-tier	ELISA+WB IgM	9.0
	Świętoszów				37			Standard two-tier	ELISA+WB IgM	2.7
	Wołów				63			Standard two-tier	ELISA+WB IgM	9.5
	Bardo				49			Standard two-tier	ELISA+WB IgG	24.4
	Legnica				51			Standard two-tier	ELISA+WB IgG	19.6
	Milicz				89			Standard two-tier	ELISA+WB IgG	12.3
	Jugów				59			Standard two-tier	ELISA+WB IgG	22.0
	Oleśnica				96			Standard two-tier	ELISA+WB IgG	16.6
	Ruszów				65			Standard two-tier	ELISA+WB IgG	15.3
	Śnieżka				48			Standard two-tier	ELISA+WB IgG	27.0
	Świeradów				55			Standard two-tier	ELISA+WB IgG	16.3
	Świętoszów				37			Standard two-tier	ELISA+WB IgG	10.8
	Wołów				63			Standard two-tier	ELISA+WB IgG	11.1
Poland	Lublin Province	Pańczuk et al. (2019)	Cross-sectional (N/A)	Not described	150	Hunters and occupationally exposed persons (agriculture, collecting groundcover fruits, recreational activity in forested areas)^d^	Range: 17–80	Standard two-tier	ELISA IgG VlsE+WB IgG and/or IgM	38.0
								Standard two-tier	ELISA IgG VlsE+WB IgG	36.7
								Standard two-tier	ELISA IgG VlsE+WB IgM	2.7
Russia	Northeastern Siberia (Sakha Republic)	Magnaval et al. (2016)	Cross-sectional (2012)	Random	77	Healthy volunteers^b^	≥18	Single tier	ELISA IgG	10.3
									WB IgG	1.6
Slovakia	Eastern Slovakia	Zákutná et al. (2015b)	Cross-sectional (2011)	Convenience	124	Blood donors^b^	≥30	Single tier	ELISA IgG	15.3
									WB IgG	1.6
Slovakia	Eastern Slovakia	Zákutná et al. (2015a)	Cross-sectional (2011–2012)	Convenience	193	Agriculture and forestry workers^d^	≥30	Single tier	ELISA IgG	29.2
					36	Police and border customs agents^d^		Single tier	ELISA IgG	11.1
					48	Persons frequently staying in the countryside^d^		Single tier	ELISA IgG	20.8
Slovakia	Senec and Senica districts	Bazovská et al. (2010)	Cross-sectional (2008–2009)	Not described	302	Blood donors^b^	Not described	Single tier	ELISA IgG+WB IgG VlsE	8.6
Slovakia	Eastern Slovakia	Bušová et al. (2018)	Cross-sectional (2013–2016)	Not described	135	Gardeners and soldiers working with occupational exposure to ticks^d^	Mean (SD): 35.76 (11.17)	Single tier	ELISA IgG	15.6
									ELISA IgM	5.9
Slovakia	Slovakia	Bazovska et al. (2005)	Cross-sectional (1987–2004)	Not described	250	Blood donors^b^	Not described	Standard two-tier	ELISA+WB IgG and/or IgM	12.8
Slovenia	Five establishments of the Slovenian Forest Service	Rojko et al. (2005)	Cross-sectional (March to November 2002)	Random	112	Forestry workers^d^	Median (range): 40 (22–62)	Single tier	ELISA IgG	25.8
									ELISA IgM	16.4
									IFA	9.8
					93	Indoor workers from the same region^b^	Median (range): 42 (23–65)	Single tier	ELISA IgG	9.7
									ELISA IgM	16.2
									IFA	4.3
Ukraine	National^b^	Biletska et al. (2008)	Cross-sectional (2003–2006)	Not described	2393	Healthy people^b^	Not described	Single tier	IFA IgG+IgM	34.3
	Ukrainian Polissya				567			Single tier	IFA IgG+IgM	32.6
	Forest steppe				498			Single tier	IFA IgG+IgM	35.3
	Steppe				967			Single tier	IFA IgG+IgM	38.2
	Carpathian region				361			Single tier	IFA IgG+IgM	25.2

^a^ Includes single-tier test results and two-tier overall test results based on a standard or modified algorithm (Branda and Steere 2021, Marques et al. 2021).

^b^ General population.

^c^ Nationally representative general population.

^d^ High-risk population.

ELISA, enzyme-linked immunoassay; IgG, immunoglobulin G; IgM, immunoglobulin M; IFA, immunofluorescence assay; N/A, not available; SD, standard deviation; SP, seroprevalence; VlsE, variable major protein-like sequence, expressed; WB, Western blot.

**Table 2.** Estimates of Lyme Borreliosis Seroprevalence in Northern Europe from Published Literature, 2005–2020

Country	National or region within country	References	Study design (data collection period)	Sampling method	Sample size, N	Cohort description	Age group, years	Diagnostic testing strategy	Type of diagnostic test	Final SP result, %^a^
Baltic States										
Estonia	Saaremaa	Parm et al. (2015)	Cross-sectional (2012)	Cluster^b^	184	Hunters^c^	Median (IQR): 41 (29–50)	Single tier	ELISA IgG	46.7
								Single tier	ELISA IgM	1.0
								Single tier	ELISA IgM+IgG	7.0
Lithuania	National^d^	Motiejunas et al. (1994)	Cross-sectional (1988)	Random	268	Foresters^c^	Not described	Single tier	IFA IgG	14.0
					115	Field workers^c^		Single tier	IFA IgG	22.0
					68	Veterinarians^c^		Single tier	IFA IgG	32.0
Scandinavia										
Finland	National^d^	Cuellar et al. (2020)	Cross-sectional (1968–1972)	Convenience	994	General population^e^	Median (range): 57 (15–86)	Modified two-tier^f^	Whole-cell sonicate IgG+C6 Lyme ELISA+RecomBead IgG	20.0
Finland	National^d^	van Beek et al. (2018)	Cross-sectional (2011)	Cluster	2000	General population^e^	≥29	Modified two-tier^g^	Whole-cell sonicate IgG+C6 Lyme ELISA+RecomBead IgG	4.3
Norway	Sogn and Fjordane	Hjetland et al. (2014)	Cross-sectional (2010)	Convenience	1213	Blood donors^e^	Mean (range): 45.8 (19–69)	Standard two-tier	ELISA IgG VlsE+WB IgG	6.1
								Standard two-tier	ELISA IgG VlsE+WB IgM	2.8
								Standard two-tier	ELISA IgM VlsE+WB IgM	4.9
								Standard two-tier	C6 ELISA+WB IgG	5.8
								Standard two-tier	C6 ELISA+WB IgM	2.3
Norway	National^d^	Vestrheim et al. (2016)	Cross-sectional (2011–2013)	Not described	3057	Residual sera (pertussis study)^e^	≥2	Single tier	ELISA IgG VlsE	2.7
								Single tier	EIA IgG	3.4
	Akershus				601			Single tier	ELISA IgG VlsE	2.0
	Akershus				601			Single tier	EIA IgG	1.8
	Oslo				547			Single tier	ELISA IgG VlsE	4.0
	Oslo				547			Single tier	EIA IgG	3.5
	Telemark				178			Single tier	ELISA IgG VlsE	3.9
	Telemark				178			Single tier	EIA IgG	4.5
	Vest-Agder				198			Single tier	ELISA IgG VlsE	7.6
	Vest-Agder				198			Single tier	EIA IgG	9.6
	Hordaland				499			Single tier	ELISA IgG VlsE	2.4
	Hordaland				499			Single tier	EIA IgG	3.8
	Sogn og Fjordane				120			Single tier	ELISA IgG VlsE	2.5
	Sogn og Fjordane				120			Single tier	EIA IgG	4.2
	Hedmark				194			Single tier	ELISA IgG VlsE	1.5
	Hedmark				194			Single tier	EIA IgG	1.0
	Nordland				239			Single tier	ELISA IgG VlsE	0.8
	Nordland				239			Single tier	EIA IgG	2.1
	Troms				180			Single tier	ELISA IgG VlsE	0
	Troms				180			Single tier	EIA IgG	1.1
	Sør-Trøndelag				301			Single tier	ELISA IgG VlsE	2.7
	Sør-Trøndelag				301			Single tier	EIA IgG	2.7
Norway	Søgne	Thortveit et al. (2020)	Cross-sectional (2015–2016)	Consecutive	2968	General population^e^	Range: 18–69	Single tier	ELISA IgG	22.9
Sweden	Kalmar County	Carlsson et al. (2018)	Cross-sectional (2012–2013)	Consecutive	873	Blood donors with no LB history^e^	Median (range): 45 (18–72)	Single tier Single tier	IFA IgG (≥8) IFA IgG (≥12)	11.0 8.0
					115	Blood donors with unknown history of LB^e^	Median (range): 48 (18–68)	Single tier	IFA IgG (≥8)	17.0
Sweden	Kalmar County	Johansson et al. (2017)	Cross-sectional (2011/2014)	Consecutive	573	Blood donors^e^	Range: 18–69	Single tier	C6 ELISA IgG and/or IgM	23.2
Scotland	Edinburgh, Southeast Scotland, West of Scotland	Munro et al. (2015)	Cross-sectional (2010–2011)	Convenience	1440	Blood donors	Not described	Standard two-tier	ELISA IgG and/or IgM+WB IgG	4.2

^a^ Includes single-tier test results and two-tier overall test results based on a standard or modified algorithm (Branda and Steere 2021, Marques et al. 2021).

^b^ Cluster sampling: methodology that involves (1) dividing the population into subgroups or clusters that are not necessarily (and preferably not) homogeneous, (2) drawing a random sample of the clusters, and (3) selecting all or a random sample of persons in each cluster.

^c^ High-risk population.

^d^ Nationally representative general population.

^e^ General population.

^f^ Sample taken ∼50 years ago and then tested with current diagnostic test.

^g^ Modified based on current guidelines for articles published after modified testing strategies were published (Branda and Steere 2021).

C6, C6 protein of the variable major protein-like sequence lipoprotein; EIA, enzyme immunoassay; IQR, interquartile range; LB, Lyme borreliosis.

**Table 3.** Estimates of Lyme Borreliosis Seroprevalence in Southern Europe from Published Literature, 2005–2020

Country	National or region within country	References	Study design (data collection period)	Sampling method	Sample size, N	Cohort description	Age group, years	Diagnostic testing strategy	Type of diagnostic test	Final SP result, %^a^
Italy	Arezzo, Florence, and Siena	Tomao et al. (2005)	Prospective cohort (1999–2001)	Not described	365	Blood donors^b^	Mean (SD): 43.36 (8.16)	Standard two-tier^c^	ELISA+WB IgG and/or IgM	3.5
								Standard two-tier^d^	ELISA+WB IgG and/or IgM	1.6
					412	Forestry workers^e^	Mean (SD): 43.71 (11.13)	Standard two-tier^c^	ELISA+WB IgG and/or IgM	7.0
								Standard two-tier^d^	ELISA+WB IgG and/or IgM	3.8
Italy	Lazio region	Di Renzi et al. (2010)	Prospective cohort (2008)	Not described	145	Forestry rangers^e^	Mean (SD): 41.0 (7.8)	Standard two-tier	ELISA+WB IgG	0.68
								Standard two-tier	ELISA+WB IgM	13.1
					282	Blood donors^b^	Mean (SD): 40.4 (9.7)	Standard two-tier	ELISA+WB IgG	1.1
								Standard two-tier	ELISA+WB IgM	8.2
Serbia	Belgrade	Jovanovic et al. (2015)	Prospective cohort (2014)	Convenience	34	Forestry workers^e^	Range: 25–45	Standard two-tier	ELISA+WB IgM and/or IgG	11.8
					35	Blood donors^b^		Standard two-tier	ELISA+WB IgM and/or IgG	8.6
Serbia	Belgrade	Krstić and Stajković (2007)	Cross-sectional (2005)	Cluster	34	Occupationally exposed to ticks (public utility workers)^e^	Not described	Single tier	ELISA IgG and/or IgM	23.5
					35	Not occupationally exposed to ticks (military medical cadets)^b^		Single tier	ELISA IgG and/or IgM	2.9
Spain	Guadalajara province	Lledo et al. (2019)	Cross-sectional (2019)	Not described	100	Occupationally exposed to ticks (forestry, agriculture, cattle raising)^e^	Median (IQR): 33 (29.5–40.25)	Single tier	IFA IgG	7.0
Spain	Asturias	Barreiro-Hurle et al. (2020)	Cross-sectional (2014)	Convenience	316	Blood donors^b^	Mean (SD): 46 (8.5)	Standard two-tier	ELISA+WB IgG	5.1
					432	Outpatients without infectious disease^b^	Mean (SD): 54 (14.1)	Standard two-tier	ELISA+WB IgG	14.4
Spain	Navarra	Oteiza-Olaso et al. (2011)	Cross-sectional (1996)	Random	1429	Residents of Navarra^b^	≥15	Single tier	ELISA C6	4.4
						Stockbreeders				13.2
						Farmers				3.5
Turkey	Erzurum Center and Pasinler district	Uyanık et al. (2009)	Prospective cohort (2007–2008)	Convenience	101	Residents with a high risk of tick exposure living in a high-risk area (Erzurum Province)^e^	Male mean: 39.5; female mean: 33.7	Modified two-tier^f^	ELISA+ELFA IgG	2.0
					79	Blood donors with low risk of exposure to ticks^b^	Male mean: 37.9; female mean: 35.8	Modified two-tier^f^	ELISA+ELFA IgG	2.5
Turkey	Düzce	Kaya et al. (2008)	Prospective cohort (2007)	Convenience	349	Forestry workers^e^	Median (range): 46.9 (14–92)	Standard two-tier	ELISA+WB IgG	1.1
					193	Blood donors^b^	≥10	Standard two-tier	ELISA+WB IgG	0.0
Turkey	Düzce	Akar et al. (2019)	Cross-sectional (2016)	Convenience	193	Residents of a high-risk area (Duzce)^e^	Mean (SD): 47.4 (13.5)	Standard two-tier	ELISA+WB IgM	1.5
								Standard two-tier	ELISA+WB IgG	6.2
Turkey	Van region	Parlak et al. (2015)	Cross-sectional (2012)	Cluster (random sampling in clusters)	446	Residents of a high-risk area (Van region)^e^	Mean (SD): 39.6 (15.5)	Standard two-tier	ELISA+WB IgG	0.9
Turkey	Tekkekoy district	Aslan Başbulut et al. (2012)	Cross-sectional (2006)	Cluster (random sampling in clusters)	419	Tekkeköy (high tick population)^e^	Mean (SD): 33.07 (19.58)	Standard two-tier	ELISA+WB IgG	3.3
Turkey	Province of Bolu	Bucak et al. (2016)	Cross-sectional (August to October 2013)	Stratified random	196	Residents of Bolu^b^	Adults and children	Standard two-tier	ELISA+WB IgG and/or IgM	8.1
Turkey	Erzincan	Cikman et al. (2019)	Cross-sectional (2014)	Cluster	368	Residents of Erzincan (high tick population)^e^	Mean (SD): 51.43 (16.91)	Standard two-tier	ELISA+WB IgG	2.1
Turkey	Sivas region	Güneş et al. (2005)	Cross-sectional (2005)	Random	270	Persons with contact with livestock^e^	Mean (range): 38.2 (13–80)	Single tier	ELISA IgG	0.4
					135	Healthy controls^b^		Single tier	ELISA IgG	0.7
Turkey	Van region	Bozkurt et al. (2008)	Prospective cohort (2008)	Random	460	Residents of the Van region^b^		Single tier	EIA IgM	5.8
									EIA IgG	1.5
	Özalp				31			Single tier	EIA IgG	6.5
	Gevaf				14			Single tier	EIA IgG	7.1
	Muradiye				29			Single tier	EIA IgG	3.4
	Van				185			Single tier	EIA IgG	1.6
	Ozalp				31			Single tier	EIA IgM	19.4
	Çaldran				32			Single tier	EIA IgM	18.8
	Baflkale				30			Single tier	EIA IgM	10.0
	Edremit				11			Single tier	EIA IgM	9.1
	Gevaf				14			Single tier	EIA IgM	7.1
	Muradiye				29			Single tier	EIA IgM	6.9
	Van				185			Single tier	EIA IgM	3.8
	Ercis				77			Single tier	EIA IgM	1.3
Turkey	Trabzon city and counties	Cora et al. (2017)	Retrospective, cross-sectional (2007–2008)	Convenience	884	Residents of Trabzon	Range: 20–79	Standard two-tier	ELISA+WB IgG	14.5
					555	Occupationally exposed to ticks^e^		Standard two-tier	ELISA+WB IgG	15.8
					329	Not occupationally exposed to ticks^b^		Standard two-tier	ELISA+WB IgG	12.2
	Akçaabat				95			Standard two-tier	ELISA+WB IgG	4.2
	Araklı				49			Standard two-tier	ELISA+WB IgG	22.4
	Çaykara				6			Standard two-tier	ELISA+WB IgG	50
	Düzköy				12			Standard two-tier	ELISA+WB IgG	0
	Maçka				22			Standard two-tier	ELISA+WB IgG	9.1
	Of				48			Standard two-tier	ELISA+WB IgG	14.6
	Sürmene				37			Standard two-tier	ELISA+WB IgG	13.5
	Vakfıkebir				44			Standard two-tier	ELISA+WB IgG	22.7
	Yomra				30			Standard two-tier	ELISA+WB IgG	26.7
Turkey	Manisa	Gazi et al. (2016)	Cross-sectional (2012)	Random	324	Farmers	Mean (SD): 49.16 (16.78)	Single tier	IFA IgG VlsE	4.3

^a^ Includes single test results and two-tier overall test results based on a standard or modified algorithm (Branda and Steere 2021, Marques et al. 2021).

^b^ General population.

^c^ According to manufacturer's instructions.

^d^ According to CDC-recommended criteria.

^e^ High-risk population.

^f^ Modified based on current guidelines for articles published after modified testing strategies were published (Branda and Steere 2021).

CDC, Centers for Disease Control and Prevention; ELFA, enzyme-linked fluorescent assay.

**Table 4.** Estimates of Lyme Borreliosis Seroprevalence in Western Europe from Published Literature, 2005–2020

Country	National or region within country	References	Study design (data collection period)	Sampling method	Sample size, N	Cohort description	Age group, years	Diagnostic testing strategy	Type of diagnostic test	Final SP result, %^a^
Austria	Districts of Burgenland	Cetin et al. (2006)	Cross-sectional (2002–2003)	Convenience	1253	Hunters^b^	Mean (SD): 51 (13)	Standard two-tier	ELISA+WB IgG	53.7
Austria	National^c^	Sonnleitner et al. (2015)	Cross-sectional (2009)	Not described	1607	Blood donors^d^	≥18	Standard two-tier	ELISA+WB IgG	5.2
	Lech Valley				58			Standard two-tier	ELISA+WB IgG	9.0
	Upper Inn Valley				120			Standard two-tier	ELISA+WB IgG	3.0
	Central Inn Valley				411			Standard two-tier	ELISA+WB IgG	6.0
	Lower Inn Valley				317			Standard two-tier	ELISA+WB IgG	10.0
	East Tyrol				104			Standard two-tier	ELISA+WB IgG	7.0
	Pustertal				95			Standard two-tier	ELISA+WB IgG	1.0
	Eisack Valley				79			Standard two-tier	ELISA+WB IgG	0
	Upper Eisack Valley				277			Standard two-tier	ELISA+WB IgG	2.0
	Lower Eisack Valley				144			Standard two-tier	ELISA+WB IgG	2.0
Belgium	Wallonia	De Keukeleire et al. (2016)	Cross-sectional (2011)	Convenience	31	Farmers^b^ Veterinarians^b^		Single tier	ELISA IgG	9.7
					96			Single tier	ELISA IgG	4.1
	Hainaut				26			Single tier	ELISA IgG	11.5
	Leige				37			Single tier	ELISA IgG	8.1
	Luxemburg				26			Single tier	ELISA IgG	3.9
Belgium	Wallonia	De Keukeleire et al. (2018)	Cross-sectional (2000–2013)	Convenience	310	Forestry workers^b^	Mean (range): 49 (24–65)	Single tier	ELISA IgG	21.6
	Namur				50			Single tier	ELISA IgG	34.0
	Arlon				24			Single tier	ELISA IgG	29.0
	Malmedy				42			Single tier	ELISA IgG	14.0
	Neufchâteau				44			Single tier	ELISA IgG	14.0
Belgium	National^c^	Lernout et al. (2019)	Cross-sectional (2013–2015)	Convenience	3215	Serum bank samples	All ages	Standard two-tier	CLIA IgG+WB IgG	1.1
	Region									
	Brussels				378			Standard two-tier	CLIA IgG+WB IgG	1.0
	Flanders				2052			Standard two-tier	CLIA IgG+WB IgG	1.3
	Wallonia				785			Standard two-tier	CLIA IgG+WB IgG	0.7
	Provinces									
	West Flanders				522			Standard two-tier	CLIA IgG+WB IgG	0.3
	East Flanders				431			Standard two-tier	CLIA IgG+WB IgG	0.4
	Flemish Brabant				532			Standard two-tier	CLIA IgG+WB IgG	1.5
	Antwerp				551			Standard two-tier	CLIA IgG+WB IgG	1.2
	Limburg				394			Standard two-tier	CLIA IgG+WB IgG	3.0
	Hainaut				298			Standard two-tier	CLIA IgG+WB IgG	0
	Walloon Brabant				69			Standard two-tier	CLIA IgG+WB IgG	2.8
	Liège				175			Standard two-tier	CLIA IgG+WB IgG	1.7
	Namur				62			Standard two-tier	CLIA IgG+WB IgG	0
	Luxembourg				181			Standard two-tier	CLIA IgG+WB IgG	0.5
France	National^c^	Thorin et al. (2008)	Cross-sectional (2002–2003)	Convenience	2975	Forestry field professionals^b^	Range: 17–81	Standard two-tier	ELISA+WB IgG and/or IgM	14.1
	Alsace				636			Standard two-tier	ELISA+WB IgG and/or IgM	26.9
	Lorraine				885			Standard two-tier	ELISA+WB IgG and/or IgM	16.5
	Champagne-Ardenne				485			Standard two-tier	ELISA+WB IgG and/or IgM	8.2
	Burgundy				400			Standard two-tier	ELISA+WB IgG and/or IgM	7.5
	Franche-Comté				554			Standard two-tier	ELISA+WB IgG and/or IgM	5.6
France	Southwestern France	Ruiz et al. (2020)	Cohort study (2007–2016)	Random	689	Retired farmers	≥65	Standard two-tier	ELISA IgG+WB IgG	6.5
Germany	National^c^	Wilking et al. (2015)	Cross-sectional (2008–2011)	Not described	6945	General population^d^	≥18	Standard two-tier	ELISA+WB IgG	10.6
	Middle states				3087			Standard two-tier	ELISA+WB IgG	9.8
	Western states				4748			Standard two-tier	ELISA+WB IgG	10.1
	Eastern states				2197			Standard two-tier	ELISA+WB IgG	11.6
	Northern states				1767			Standard two-tier	ELISA+WB IgG	10.2
Germany	National^c^	Dehnert et al. (2012)	Cross-sectional (2003–2006)	Not described	12,614	Volunteers^d^	1–17	Standard two-tier	ELISA+WB IgG	3.6
	West				8248			Standard two-tier	ELISA+WB IgG	4.0
	East				4272			Standard two-tier	ELISA+WB IgG	4.2
	North				3294			Standard two-tier	ELISA+WB IgG	3.6
	Central				5522			Standard two-tier	ELISA+WB IgG	3.7
	South				3704			Standard two-tier	ELISA+WB IgG	5.1
	Rural area				2745			Standard two-tier	ELISA+WB IgG	5.1
	Small town				3322			Standard two-tier	ELISA+WB IgG	4.6
	Mid-size town				3666			Standard two-tier	ELISA+WB IgG	3.7
	Metropolitan area				2787			Standard two-tier	ELISA+WB IgG	3.0
Netherlands	National^c^	van Gorkom et al. (2017)	Cross-sectional (2013–2015)	Convenience	147	Healthy individuals^d^	Mean (IQR): 42.3 (28.0–53.4)	Standard two-tier	ELISA C6+WB IgG and/or IgM	13.6

^a^ Includes single-tier test results and two-tier overall test results based on a standard or modified algorithm (Branda and Steere 2021, Marques et al. 2021).

^b^ High-risk population.

^c^ Nationally representative general population.

^d^ General population.

CLIA, chemiluminescent immunoassay.

As a secondary analysis, to explore the impact of risk status on seroprevalence, seroprevalence odds ratios (ORs) and corresponding 95% CIs were calculated in R using the approximate Bayesian CIs (Laud 2021). The OR was calculated as seroprevalence of positive serologic test results in the risk group under study (high risk of exposure to ticks) compared with the seroprevalence in a control group (low or unknown risk of exposure to ticks) (Supplementary Table S2). Criteria used to classify study participants by their risk of exposure to ticks is defined in Supplementary Table S5. ORs >1 (with CIs also >1) indicate significantly higher seroprevalence among the high-risk group compared with the low-risk group.

## Results

### Search results

**FIG. 2.**
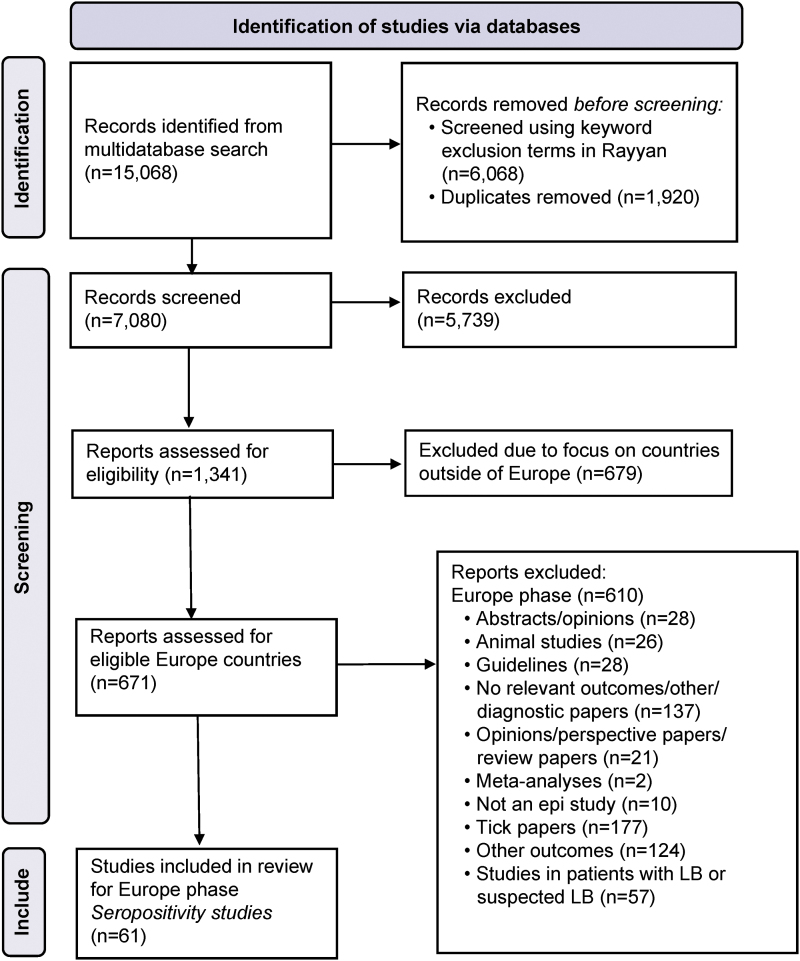
PRISMA flow diagram. PRISMA, Preferred Reporting Items for Systematic Reviews and Meta-Analyses.

**FIG. 3.**
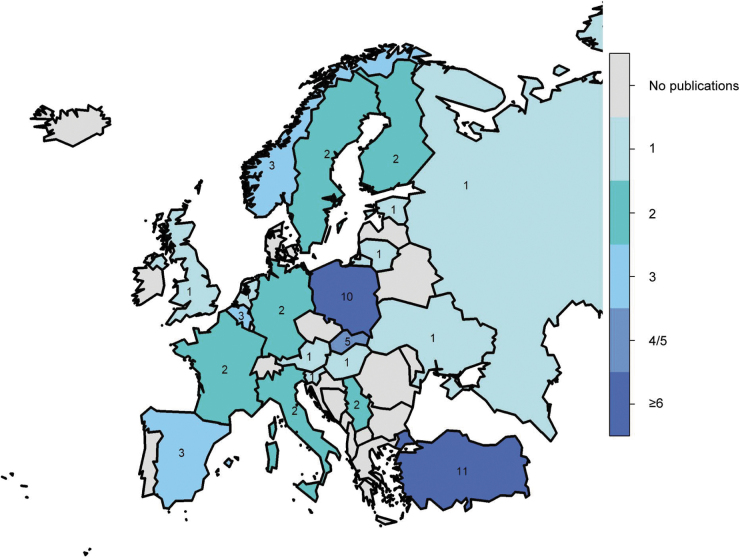
Heat map of number of publications by country from literature published between 2005 and 2020. *N* = 61.

We included 61 articles from 22 European countries for analysis (Fig. 2); of these countries, seven had only 1 study, while Poland had 10 articles and Turkey had 11. The number of publications by country is shown in a heat map (Fig. 3). Fifty-two studies used a cross-sectional design, eight used a prospective cohort design, and one used a retrospective cohort design. Some studies contained both groups, general and high-risk populations (Supplementary Table S3).

A summary of the included articles with reported seroprevalence estimates and corresponding diagnostic tests and strategies is provided (Tables 1–4). The results are organized by European Region per World Health Organization (WHO) Regional Classifications (Supplementary Table S4) (World Health Organization 2022).

### Study populations

Of the 61 total studies, 39 (64%) were population based, with blood samples collected from the general population (Supplementary Table S3), including office workers, healthy volunteers, and patients without clinical suspicion of LB, while 34 (56%) studies collected blood samples from populations with high risk of exposure to ticks (Supplementary Tables S3 and S5), including hunters, forestry and field workers, rangers, veterinarians, farmers and retired farmers, and soldiers. Among the 39 population-based studies, 14 collected nationally representative blood samples from the general population. Some studies were conducted among both general and high-risk groups, so there was overlap (*N* > 61).

### Diagnostic testing strategies

Diagnostic testing methods and strategies varied among studies (Tables 1–4). Many studies used more than one test. Of the 61 studies, 28 used single-tier testing (23 used ELISA or EIA; 5 used IFA), and 30 studies utilized standard two-tier testing (ELISA with WB [IgG, IgM, or both]). Eight articles reported seroprevalence as a combined result using IgM and/or IgG detected by WB, and three studies used modified two-tier testing, including two studies from Finland (whole-cell sonicate IgG+C6 [C6 protein of the variable major protein-like sequence lipoprotein] Lyme ELISA+IFA IgG) and one study from Turkey (ELISA+enzyme-linked IFA [IgG]).

Five of the 61 studies used the VlsE-based EIA as either single-tier testing or part of a two-tier method. This included two studies in Norway (Hjetland et al. 2014, Vestrheim et al. 2016), one in Slovakia (Bazovská et al. 2010), one in Turkey (Gazi et al. 2016), and one in Poland (Pańczuk et al. 2019).

Many studies tested blood samples collected from participants in more than one period. Tests included both commercial (49 studies) and in-house (3 studies) assays; 8 studies did not give information on the source.

### Seroprevalence estimates by European region

#### Eastern Europe (Czech Republic, Hungary, Poland, Russia, Slovakia, Slovenia, Ukraine)

##### Seroprevalence among general populations

Twenty-three articles reported seroprevalence of LB in seven Eastern European countries (Table 1) (Bazovska et al. 2005, Rojko et al. 2005, Hajek et al. 2006, Biletska et al. 2008, Cisak et al. 2008, Buczek et al. 2009, Bazovská et al. 2010, Podsiadly et al. 2011, Lakos et al. 2012, Machcińska et al. 2013, Kocbach and Kocbach 2014, Tokarska-Rodak et al. 2014, Zákutná et al. 2015a, 2015b, Kuchynka et al. 2016, Magnaval et al. 2016, Zając et al. 2017, Bura et al. 2018, Bušová et al. 2018, Kiewra et al. 2018, Kříž et al. 2018, Pańczuk et al. 2019, Pawelczyk et al. 2019). National seroprevalence estimates using a single-tier testing strategy on healthy persons and/or indoor workers were 5% in Poland (ELISA IgG, healthy blood donors) (Pawelczyk et al. 2019), 9.7% in Slovenia (ELISA IgG) (Rojko et al. 2005), 10.3% in Russia (ELISA IgG) (Magnaval et al. 2016), and 34.3% in Ukraine (IFA IgG+IgM) (Biletska et al. 2008).

##### Seroprevalence in high-risk groups

The seroprevalence of LB in populations with high-risk occupations using standard two-tier testing was 17.8% (ELISA+WB IgG) in forestry workers in Świętoszów, Poland (Kiewra et al. 2018) and 38% (ELISA IgG VlsE+WB IgG and/or IgM) in hunters and occupationally exposed persons in Lublin Province, Poland (Pańczuk et al. 2019). In studies with single-tier testing, the seroprevalence in forestry workers was 25.8% (ELISA IgG) in Slovenia (Rojko et al. 2005), 37.2% (ELISA IgG+IgM) in Hungary (Lakos et al. 2012), and 29.2% (ELISA IgG) in Slovakia.

##### Subnational seroprevalence

Subnational data from five regions in Ukraine were available from a study that assessed seroprevalence in healthy persons based on a single-tier testing strategy (IFA IgM+IgG) (Table 1). Seroprevalence ranged from 25.2% in the Carpathian region to 38.2% in the Steppe and up to 70% in the local administrative district of Kiverci in the region of Volyn Oblast (Biletska et al. 2008).

#### Northern Europe (Estonia, Finland, Lithuania, Norway, Scotland, Sweden)

##### Seroprevalence among general populations

Seven articles reported LB seroprevalence in Finland, Norway, and Sweden in the general population (Table 2) (Hjetland et al. 2014, Vestrheim et al. 2016, Johansson et al. 2017, Carlsson et al. 2018, van Beek et al. 2018, Cuellar et al. 2020, Thortveit et al. 2020), with testing based on samples from blood donors and residual sera from clinical trials and population-based surveys. General seroprevalence estimates were 6.1% (ELISA IgG VlsE+WB IgG) and 5.8% (C6 ELISA+WB IgG) among healthy blood donors in Norway (Hjetland et al. 2014). Among the general population in Finland, LB seroprevalence was 4.3% using modified two-tier testing (van Beek et al. 2018) versus 20% using a three-step testing method (as part of a modified two-tier testing strategy) in serum samples collected between 1968 and 1972 (Cuellar et al. 2020).

##### Seroprevalence in high-risk groups

Two studies of high-risk groups that used single-tier testing strategies (Motiejunas et al. 1994, Parm et al. 2015) reported seroprevalence estimates of 46.7% (ELISA IgG) in Estonian hunters and 14–32% (IFA IgG) in Lithuanian forestry workers, outdoor field workers, and veterinarians (Table 2).

##### Subnational seroprevalence

Five studies reported seroprevalence estimates from regions within Norway and Sweden. In Norway, seroprevalence ranged from 0% to 22.9% using single-tier testing strategies and various testing methods (Thortveit et al. 2020, Vestrheim et al. 2016) (Table 2). In Sweden, LB seroprevalence in Kalmar County in the southeast using single-tier testing (IFA IgG) was estimated to be between 8% and 17% depending on the assay cutoff employed and blood donor population studied (Carlsson et al. 2018). One study (Munro et al. 2015) reported a seroprevalence of 4.2% in blood donors by a standard two-tier testing method in the regions of West of Scotland, Edinburgh, and South East Scotland (Table 2).

#### Southern Europe (Italy, Serbia, Spain, Turkey)

##### Seroprevalence among general populations

Eighteen articles reported seroprevalence of LB in four countries in Southern Europe (Güneş et al. 2005, Tomao et al. 2005, Krstić and Stajković 2007, Bozkurt et al. 2008, Kaya et al. 2008, Uyanık et al. 2009, Di Renzi et al. 2010, Oteiza-Olaso et al. 2011, Aslan Başbulut et al. 2012, Jovanovic et al. 2015, Parlak et al. 2015, Bucak et al. 2016, Gazi et al. 2016, Cora et al. 2017, Akar et al. 2019, Cikman et al. 2019, Lledo et al. 2019, Barreiro-Hurle et al. 2020). All studies reported subnational rather than country-wide estimates (Table 3); 15 reported seroprevalence in groups at high risk of tick exposure based on occupation or residential area, and 11 reported on populations at low risk.

Estimates of LB seroprevalence were <10% for the general population in most regions of Italy, Serbia, Spain, and Turkey. An exception to this, in Turkey, determined using two-tier testing (ELISA+WB IgG), was an overall seroprevalence of 14.5% in residents of the city and environs of Trabzon, including Araklı (22.4%), Maçka (9.1%), Of (14.6%), Sürmene (13.5%), Vakfıkebir (22.7%), and Yomra (26.7%) (Cora et al. 2017). Furthermore, seroprevalence determined using a single-tier strategy was reported to be 10.0–19.4% in residents of three areas in the Van region (Ozalp, Çaldran, and Baflkaleup) (Bozkurt et al. 2008).

##### Seroprevalence in high-risk groups

In high-risk populations, LB seroprevalence was <1% using standard two-tier testing (ELISA+WB IgG) both in forestry rangers in Lazio, Italy (Di Renzi et al. 2010) and in residents of the Van region of Turkey (Parlak et al. 2015), but was 23.5% in public utility workers in Belgrade, Serbia (Krstić and Stajković 2007). In low-risk populations in Southern Europe, seroprevalence using standard two-tier testing (measured by different EIAs+WB IgG and/or IgM) ranged from <1% in Lazio, Italy (Di Renzi et al. 2010) and the Düzce, Van, Sivas, and Düzköy regions of Turkey (Güneş et al. 2005, Kaya et al. 2008, Parlak et al. 2015, Cora et al. 2017) to 50% in Çaykara, Turkey (Cora et al. 2017).

#### Western Europe (Austria, Belgium, France, Germany, the Netherlands)

##### Seroprevalence among general populations

Ten articles reported estimates of LB seroprevalence in countries in Western Europe, as summarized in Table 4 (Cetin et al. 2006, Thorin et al. 2008, Dehnert et al. 2012, Sonnleitner et al. 2015, Wilking et al. 2015, De Keukeleire et al. 2016, 2018, van Gorkom et al. 2017, Lernout et al. 2019, Ruiz et al. 2020). Six articles reported seroprevalence in the general population and five reported seroprevalence in groups at high risk of occupational tick exposure. The national seroprevalence of LB in volunteers or blood donors was 3.6% (ELISA+WB IgG) in Germany (Dehnert et al. 2012) and 1.1% (chemiluminescent immunoassay [CLIA] IgG+WB IgG) in Belgium (Lernout et al. 2019) based on a two-tier testing strategy.

As observed in other parts of Europe, there was substantial heterogeneity in seroprevalence of antibodies against *Bbsl* within individual countries according to region and occupational exposure. In Austria, seroprevalence among blood donors ranged from 0% in the Eisack Valley to 10% in the Lower Inn Valley (ELISA+WB IgG) based on two-tier testing (Sonnleitner et al. 2015).

##### Seroprevalence in high-risk groups

The seroprevalence of LB in populations with high-risk occupations was 14.1% among a nationally representative cohort of forestry field professionals in France, and 53.7% among hunters in Austria (ELISA+WB IgG), using two-tier testing (Cetin et al. 2006). In Wallonia, Belgium, seroprevalence estimates based on single-tier testing (ELISA IgG) among farmers and veterinarians was 11.54% in Hainaut and 9.68% in Wallonia (De Keukeleire et al. 2016), whereas in forestry workers it reached 34% in Namur and 21.6% in Wallonia (De Keukeleire et al. 2018).

#### Seroprevalence estimates by European region and diagnostic strategy

##### Eastern Europe

The weighted mean seroprevalence of LB in Eastern Europe among studies that used (standard) two-tier testing strategies in general populations was 11.1% (Table 5). Five studies (Bazovska et al. 2005, Cisak et al. 2008, Tokarska-Rodak et al. 2014, Kuchynka et al. 2016, Pańczuk et al. 2019) used the IgG and IgM standard two-tier test; the weighted mean of the high-risk group was higher (40.6%) than in the low-risk group (11.1%). Four of 23 studies (Podsiadly et al. 2011, Kocbach and Kocbach 2014, Bura et al. 2018, Kiewra et al. 2018) measured IgG in high-risk populations only, giving a weighted mean of 24.7%. When stratified by single-tier testing (IgM and IgG), seroprevalence was higher in high-risk groups (37.2%) than low-risk groups (34.7%). For IgG alone, it was 16.1% in high-risk groups versus 12.6% in low-risk groups.

**Table 5.** Weighted Mean Lyme Borreliosis Seroprevalence in Eastern Europe from Published Literature, 2005–2020

Diagnostic testing strategy	Antibody type	Risk group	Mean SP (95% CI), %
Single tier	IgG+IgM	Low risk	34.7 (32.8–36.6)
	IgG		12.5 (9.4–16.7)
	IgM		10.7 (7.3–15.2)
	IgG+IgM	High risk	37.2 (35.2–39.2)
	IgG		16.1 (14.3–18.1)
	IgM		12.6 (11.3–14.2)
Standard two-tier	IgG+IgM	Low risk	11.1 (7.1–17.2)
	IgG+IgM	High risk	40.6 (33.6–48)
	IgG		24.7 (20.6–29.2)
	IgM		7.6 (5.6–10.3)

CI, confidence interval.

##### Northern Europe

The weighted mean seroprevalence of LB in Northern Europe among studies that used (standard) two-tier testing strategies in general populations was 4.2% (Table 6). Additionally, two studies from Finland (van Beek et al. 2018, Cuellar et al. 2020) used a modified algorithm in low-risk groups, yielding a weighted mean seroprevalence of 9.5%. Different algorithms were evaluated in this region, including one study in Norway (Hjetland et al. 2014) using a standard two-tier test with a weighted mean of 4.7% in the low-risk group. When stratified by single-tier testing, the weighted mean seroprevalence for IgG was higher in high-risk groups (26.9%) than low-risk groups (12.0%).

**Table 6.** Weighted Mean Lyme Borreliosis Seroprevalence in Northern Europe from Published Literature, 2005–2020

Diagnostic testing strategy	Antibody type	Exposure group	Mean SP (95% CI), %
Single tier	IgG+IgM	Low risk	23.2 (20.3–26.3)
	IgG		12.0 (10.9–13.2)
	IgG+IgM	High risk	7.1 (4.2–11.0)
	IgG		26.9 (21.6–2.9)
	IgM		1.1 (0.2–3.4)
Standard two-tier	IgG+IgM	Low risk	4.2 (3.3–5.5)
	IgG		4.7 (3.8–5.8)
	IgM		3.8 (3.0–4.9)
Modified two-tier	IgG	Low risk	9.5 (8.3–10.8)

##### Southern Europe

The weighted mean seroprevalence of LB in Southern Europe among studies that used (standard) two-tier testing strategies in general populations (Tomao et al. 2005, Kaya et al. 2008, Di Renzi et al. 2010, Aslan Başbulut et al. 2012, Jovanovic et al. 2015, Parlak et al. 2015, Bucak et al. 2016, Cora et al. 2017, Akar et al. 2019, Cikman et al. 2019, Barreiro-Hurle et al. 2020) was 3.9% (Table 7). The standard IgM and IgG weighted mean seroprevalence was 5.7% in high-risk groups versus 3.9% in low-risk groups. For single-tier testing (IgG and IgM), the mean seroprevalence was 8.9% in high-risk groups versus 4.4% in low-risk groups (Güneş et al. 2005, Krstić and Stajković 2007, Bozkurt et al. 2008, Oteiza-Olaso et al. 2011, Gazi et al. 2016, Lledo et al. 2019).

**Table 7.** Weighted Mean Lyme Borreliosis Seroprevalence in Southern Europe from Published Literature, 2005–2020

Diagnostic testing strategy	Antibody type	Exposure group	Mean SP (95% CI), %
Single tier	IgG+IgM	Low risk	4.4 (3.5–5.7)
	IgG		1.3 (0.6–3.0)
	IgM		5.9 (4.1–8.0)
	IgG+IgM	High risk	8.5 (7.3–10.0)
	IgG		3.2 (1.7–5.6)
Standard two-tier	IgG+IgM	Low risk	3.9 (2.2–6.6)
	IgG		10.2 (8.3–12.6)
	IgM		8.2 (5.6–11.4)
	IgG+IgM	High risk	5.7 (3.8–8.3)
	IgG		5.3 (4.0–7.3)
	IgM		6.5 (4.0–10.3)
Modified two-tier	IgG	Low risk	2.5 (0.4–7.8)
	IgG	High risk	2.0 (0.3–6.1)

##### Western Europe

The weighted mean seroprevalence of LB in Western Europe among studies that used (standard) two-tier testing strategies in general populations was 13.6% (Dehnert et al. 2012, Sonnleitner et al. 2015, Wilking et al. 2015, Lernout et al. 2019) (Table 8). This was lower than the mean seroprevalence of high-risk groups (14.1%) based on one study in France (Thorin et al. 2008) and two studies that assessed seroprevalence by two-tiered IgG testing (37%) (Cetin et al. 2006, Ruiz et al. 2020). Two studies in Belgium (De Keukeleire et al. 2016, 2018) that used single-tier IgG testing in high-risk populations gave a weighted mean seroprevalence of 16.9%.

**Table 8.** Weighted Mean Lyme Borreliosis Seroprevalence in Western Europe from Published Literature, 2005–2020

Diagnostic testing strategy	Antibody type	Exposure group	Mean SP (95% CI), %
Single tier	IgG	High risk	16.9 (13.1–22.0)
Standard two-tier	IgG+IgM	Low risk	13.6 (9.2–19.1)
	IgG		5.4 (5.0–5.8)
	IgG+IgM	High risk	14.1 (13.0–15.2)
	IgG		37.0 (34.9–39.1)

#### Seroprevalence estimates by age and sex

##### Overall Europe

Seroprevalence results stratified by age group and sex are presented in Supplementary Table S1.

##### Age

Thirteen studies reported age-stratified LB seroprevalence; all observed increasing seroprevalence for antibodies against *Bbsl* with age. In adults, a seroprevalence of at least 20% was observed in older age groups: ≥50 years of age in Finland (Cuellar et al. 2020), ≥55 years of age in France (Thorin et al. 2008), ≥60 years of age in Norway and Turkey (Hjetland et al. 2014, Cora et al. 2017), and ≥70 years of age in Germany and Poland (Wilking et al. 2015, Zając et al. 2017). The highest seroprevalence was observed in Austrian hunters, in whom 59.3% of 50- to 59-year-olds and 83.3% of >70-year-olds were seropositive (Cetin et al. 2006).

One study reported seroprevalence rates by age group in children ≥2 years of age using sera collected during a pertussis vaccine study in Norway. The seroprevalence (single tier, IgG) was 3.6% in 2- to 4-year-olds, 4.1% in 5- to 9-year-olds, and 2.1% in 10- to 19-year-olds versus 3.7% in 20- to 39-year-olds and 6.0% in >50-year-olds (Vestrheim et al. 2016).

##### Sex

In 17 studies that reported seroprevalence by sex, there was a trend toward higher seroprevalence in men than women (Supplementary Table S1) regardless of risk cohort.

Among high-risk groups in Estonia, seroprevalence (ELISA IgG) in male hunters was 52.6% versus 18.7% in female hunters (ELISA IgG) (Parm et al. 2015). The seroprevalence estimate was 6.4% in male Belgian farmers and veterinarians versus 0.0% in women (ELISA IgG) (De Keukeleire et al. 2018), 16.5% in male Polish farmers versus 11.7% in women (ELISA IgG) (Zając et al. 2017), and 14.3% in male forest and field professionals in France versus 3.4% in women (ELISA+WB IgG and/or IgM) (Thorin et al. 2008).

Similar trends were observed in low-risk groups in most countries. In Spain and Norway, the seroprevalence estimates were 6.5% (ELISA+WB IgG) and 13.0% (ELISA IgG VlsE) in male blood donors versus 5.5% and 3.1% in female blood donors, respectively (Hjetland et al. 2014, Barreiro-Hurle et al. 2020). The seroprevalence in the general population of Germany was 15.0% in men and 6.6% in women (ELISA+WB IgG) (Wilking et al. 2015). In contrast, the trend was reversed in Turkey and the United Kingdom, where seroprevalence was consistently higher in women than in men. While one study reported a seroprevalence of 6.1% in male residents of a high-risk area versus 2.5% in women (ELISA+ELFA [enzyme-linked fluorescent assay] IgG) (Uyanık et al. 2009), six other studies of residents in high-risk areas reported higher seroprevalence in women, with a difference between sexes of 0.2% to 6.0% (Aslan Başbulut et al. 2012, Parlak et al. 2015, Bucak et al. 2016, Cora et al. 2017, Akar et al. 2019, Cikman et al. 2019).

None of the studies showed a statistically significant difference between sex, except for one study in high-risk residents in Turkey, including ELISA and WB test results showing more statistically significant (*p* < 0.05) greater positive results in females (5.2%) than males (0.7%; ELISA IgG) (Parlak et al. 2015).

#### Seroprevalence estimates by risk groups

The high heterogeneity of the data (*I*^2^ > 90%, even after subanalyses by diagnostic methods and strategies, age, and sex) prevented a meta-analysis, but we did explore analysis by risk group. Among studies that analyzed persons with greater risk for tick exposure, LB seroprevalence (using standard two-tier for IgG+IgM) was higher among these groups than in the general population. While the difference in Eastern Europe was statistically significant (40.6% [33.6–48.0%] vs. 11.1% [7.1–17.2%] (Supplementary Table S5) where the two 95% CI did not overlap), in Southern Europe and Western Europe, we observed higher LB seroprevalence in high-risk group compared with low-risk group but this difference was not statistically significant (95% CI overlapped).

There were 12 studies that allowed calculation of ORs to evaluate whether tick exposure risk group was associated with seroprevalence (Supplementary Tables S2 and S3) by comparison with populations at low risk of exposure to ticks. Seven reported that the odds of LB seropositivity were statistically significantly increased by 2- to 21-fold in high-risk populations, suggesting that high-risk groups, including forestry workers and rural residents and workers, share a greater risk of seropositivity compared with low-risk populations. However, CIs were wide for many of the estimates.

## Discussion

This review provides a comprehensive overview of the seroprevalence of LB in Europe. The main finding of our analysis is that LB is a disease of place (local setting) and activity (both occupational and leisure). National-level seroprevalence estimates may ignore potentially much higher subnational disease burdens, and conversely, subnational higher-risk areas do not necessarily indicate high national disease burden. Overall, LB seroprevalence estimates ranged from 0% to 70% (the latter in an administrative region in the Ukraine) (Biletska et al. 2008). There was a general trend toward higher seroprevalence for general population data in Western Europe and Eastern Europe, but on closer inspection there was substantial variation within the countries in Southern and Northern Europe; this may also be due to differences in *Bbsl* genospecies and *I. ricinus* abundance and distribution (Strnad et al. 2017, Van den Wijngaard et al. 2017, Estrada-Pena et al. 2018).

LB seroprevalence trended higher, although inconsistently, in persons undertaking at-risk occupational or leisure activities in some studies, with the highest estimate (83.3%) among hunters >70 years of age in Austria (Cetin et al. 2006) The seroprevalence in people with higher exposure to ticks was higher among these groups than in the general population (40.6% vs. 3.9%); the same occurs in a systematic review in human population where the high-risk population shows a seroprevalence of 18.8% (95% CI 10.1–29.4%) compared with a much lower seroprevalence in the general population of 5.7% (95% CI 4.3–7.3%) (Dong et al. 2022).

A 15-study meta-analysis reported that the OR for seroprevalence was 1.9 (95% CI: 1.2–3.2) worldwide in outdoor workers compared with controls (Magnavita et al. 2022). Interestingly, the risk changed over time and apparently decreased after 2010, possibly due to better education and/or occupational protection, but this trend was not consistently observed herein. A recent systematic review of global seroprevalence data and characteristics of *B. burgdorferi* in human populations (Dong et al. 2022) estimated seroprevalence to be 20.7% in Central Europe and 13.5% in Western Europe.

That review used a longer search time frame (1999–2021), whereas we captured additional studies in Europe and were ultimately more comprehensive. We also present diagnostic testing methods and strategies used, as well as our calculations of final two-tier test results, to enhance the interpretation of seroprevalence results while considering the limitations of single-tier testing (*e.g.,* more false positives). The review by Dong and colleagues was also limited by including population cohorts suspected to have LB, which we excluded herein to reduce selection bias in seroprevalence estimates and ensure that we captured estimates in populations without existing LB.

We found that LB seroprevalence increased with age, reflecting both the cumulative risk of exposure to an infected tick over time and the persistence of antibodies to *Bbsl*, which show varying and unpredictable kinetics but may persist for years or even decades after acute infection (Kalish et al. 2001, Peltomaa et al. 2003, Glatz et al. 2006). Among 61 studies, only one study demonstrated statistically significant differences between sexes, with higher positive results in females in a high-risk region of Turkey (Parlak et al. 2015).

A key data gap in the epidemiology of LB is the true percentage of infections that are symptomatic versus asymptomatic. This information is needed to compare the number of symptomatic LB cases derived from seroprevalence data with the number of surveillance-reported LB cases to estimate the under-ascertainment of symptomatic LB cases by public health surveillance. However, the proportion of asymptomatic infections is difficult to estimate given the lack of prospective studies measuring the incidence of LB.

Nevertheless, three studies in Europe have estimated the proportion of asymptomatic infected persons by measuring seroconversion following a tick bite and the development of clinical signs and symptoms of LB. The median proportion of asymptomatic infected persons from these studies was 50% (Hofhuis et al. 2013, Wilhelmsson et al. 2016, Markowicz et al. 2021). A large, placebo-controlled study of a Lyme vaccine in the United States tested blood samples in intervals of months after vaccination. In this population, asymptomatic *Bbsl* infections were recorded in 11% of participants over the study period, which dropped to 7% when adjusted for those who met the criteria for LB (Steere et al. 2003).

However, differences between the proportions of asymptomatic infections in the United States and Europe may be related to differences in study design, duration of follow-up, diagnostic criteria, timing of postinfection treatment, antibody waning, and circulating genospecies. More data are needed to understand the proportion of asymptomatic LB infections, whether different genospecies vary in virulence, and the impact of age, sex, and other factors on susceptibility to clinical disease.

Our review has several limitations. Variations in study design, populations sampled, time frame, sample size, and diagnostic methods and strategies limit data interpretation and comparability between studies. First, the articles we reviewed encompassed countries of high and low endemicity and groups at differing risk of exposure to ticks among the general population. For example, categorization of a sample as high risk based on occupation may be inaccurate if the density of infected ticks is not substantial in the region where the study was conducted. Furthermore, seroprevalence data provide an estimate of the prevalence of infection, but these results need to be qualified based on testing accuracy and the duration of antibodies. Seropositivity for antibodies to *Bbsl* reflects the prevalence of prior or current *Bbsl* infection but is not necessarily equivalent to clinical disease because not all infections are symptomatic. Hence, seroprevalence may overestimate the proportion of true clinical burden of the population that has had symptomatic LB (Hammers-Berggren et al. 1994).

Immunoassays can yield false-positive results due to detection of crossreacting antibodies to unrelated antigens from bacterial, viral, parasitic, and some inflammatory conditions (Branda and Steere 2021). The lack of a gold standard test has significantly hampered the standardization of laboratory tools to diagnose LB. Available assays may use whole-cell or one or more recombinant antigens from a variety of *Bbsl* species and show marked variability in sensitivity and specificity (Kodym et al. 2018). Studies using single-tier testing are thus prone to overestimation of LB seroprevalence (Branda and Steere 2021). Additionally, heterogeneity between studies in terms of antibody testing strategies and methods (including commercial vs. in-house) limits the extent of meaningful comparisons.

Our review contributes to our understanding of the epidemiology of LB in Europe and highlights shortfalls and impediments to understanding the disease burden. Standardized LB testing strategies to monitor seroprevalence are needed to facilitate geographic and temporal data comparisons. LB is a disease of place and needs to be monitored in such a way that localized differences in tick exposure risk and burden of disease can be detected and reported.

This study complements another systematic literature review that evaluated the incidence of LB in European countries (Burn et al. 2023 in this issue) and provides information for some countries, such as Turkey, for which incidence data are not currently available. Although the ECDC started surveillance on Lyme neuroborreliosis in 2018, studies measuring the prevalence of antibodies to *Bbsl* infection in European populations in some countries are currently the only available source to monitor LB exposure and estimate disease burden (European Center for Disease Prevention and Control 2018). Seroprevalence, by providing a measure of the prevalence of *Bbsl* infection, provides data that can be used to estimate exposure to *Bbsl* among populations.

Our study provides an up-to-date picture of LB seroprevalence estimates in European countries. Seroprevalence is also an important component of the body of information that could guide intervention strategies, because monitoring the disease burden at the local level is a necessity, particularly in countries where LB is emerging.

## Supplementary Material

Supplemental data

## Data Availability

All results of this systematic literature review are derived from the published literature as referenced.
