# Supplementary material for: Seroprevalence of Lyme Borreliosis in Europe: Results from a Systematic Literature Review (2005–2020)
Source: Vector Borne Zoonotic Dis. 2023 Apr 12;23(4):195–220. doi: 10.1089/vbz.2022.0069 (PMC10122246; doi:10.1089/vbz.2022.0069)
Supplement: Supplemental data [file Supp_TableS1-S5.docx]

**Supplement**

**Seroprevalence of Lyme Borreliosis (LB) in European countries: a systematic literature review**

**Table S1. Seroprevalence estimates of Lyme Borreliosis (antibodies to *B. burgdorferi s.l.* complex) in Europe by age, sex, and region**

| **Country** | **Reference** | **Study design**  **(Year of data collection)** | **Sampling method** | **Sample size (N)** | **Cohort**  **Description** | **Age group (years)** | **Sex** | **Diagnostic Testing Strategy** | **Type of diagnostic test(s)** | **Final SP Result % (95% CI) ^a^** |  |
| --- | --- | --- | --- | --- | --- | --- | --- | --- | --- | --- | --- |
| **Baltic States** | |  |  |  |  |  |  |  |  |  |  |
| Estonia | Parm 2015 ^1^ | Cross-sectional Survey (2012) | Cluster ^b^ | 184 | Hunters | Median (IQR): 41 (29-50) | Both | Single Tier | ELISA IgG | 46.7 (39.6-53.9) |  |
|  |  |  |  |  |  |  |  | Single Tier | ELISA IgM | 1.0 (0.3-3.7) |  |
|  |  |  |  |  |  |  |  | Single Tier | ELISA IgM+ IgG | 7.0 (4.1-11.6) |  |
|  |  |  |  | 152 |  |  | Male | Single Tier | ELISA IgG | 52.6 (44.7-60.4) |  |
|  |  |  |  |  |  |  |  | Single Tier | ELISA IgM | 5.9 (3.1-10.8) |  |
|  |  |  |  | 32 |  |  | Female | Single Tier | ELISA IgG | 18.7 (8.9-35.3) |  |
|  |  |  |  |  |  |  |  | Single Tier | ELISA IgM | 3.1 (0.6-15.7) |  |
| **Scandinavia** |  |  |  |  |  |  |  |  |  |  |  |
| Finland | Cuellar 2019 ^2^ | Cross sectional (1968-1972) | Convenience | 994 | General population | Median (range): 57 (15-86) | Both | Modified 2-Tier ^c^ | Whole cell sonicate IgG+ C6 Lyme ELISA + RecomBead IgG | 20 (17.6-22.6) |  |
|  |  |  |  |  |  |  |  |  |  |  |  |
|  |  |  |  |  |  |  |  |  |  |  |  |
|  |  |  |  | 44 |  | 15-29 |  | Modified 2-Tier |  | 4.5 (1.2-15.1) |  |
|  |  |  |  | 68 |  | 30-39 | Both | Modified 2-Tier |  | 8.8 (4.1-17.9) |  |
|  |  |  |  | 186 |  | 40-49 | Both | Modified 2-Tier |  | 13.9 (9.7-19.6) |  |
|  |  |  |  | 291 |  | 50-59 | Both | Modified 2-Tier |  | 23.3 (18.8-28.5) |  |
|  |  |  |  | 253 |  | 60-69 | Both | Modified 2-Tier |  | 22.9 (18.2-28.5) |  |
|  |  |  |  | 152 |  | >70 | Both | Modified 2-Tier |  | 25.6 (19.3-33.1) |  |
| Norway | Hjetland 2014 ^3^ | Cross sectional (2010) | Convenience | 1213 | Blood donors | Mean (range): 45.8 (19–69) | Both | Standard 2-Tier | ELISA IgG VlsE+ WB IgG | 6.1 (4.9-7.6) |  |
|  |  |  |  |  |  |  |  | Standard 2-Tier | ELISA IgG VlsE+ WB IgM | 2.8 (2-3.9) |  |
|  |  |  |  |  |  |  |  | Standard 2-Tier | ELISA IgM VlsE+ WB IgM | 4.9 (3.8-6.3) |  |
|  |  |  |  |  |  |  |  | Standard 2-Tier | C6 ELISA+ WB IgG | 5.8 (4.6-7.3) |  |
|  |  |  |  |  |  |  |  | Standard 2-Tier | C6 ELISA+ WB IgM | 2.3 (1.6-3.3) |  |
|  |  |  |  | 544 |  |  | Female | Standard 2-Tier (first step) | ELISA IgG VlsE | 5.5 (3.9-7.8) |  |
|  |  |  |  |  |  |  |  |  | ELISA IgM VlsE | 7.9 (5.9-10.5) |  |
|  |  |  |  | 669 |  |  | Male | Standard 2-Tier (first step) | ELISA IgG VlsE | 13.0 (10.7-15.8) |  |
|  |  |  |  |  |  |  |  |  | ELISA IgM VlsE | 8.4 (6.5-10.8) |  |
|  |  |  |  | 80 |  | 19-29 | Both | Standard 2-Tier (first step) | ELISA IgG VlsE | 2.5 (0.7-8.7) |  |
|  |  |  |  |  |  |  |  |  | ELISA IgM VlsE | 5.0 (2-12.2) |  |
|  |  |  |  | 235 |  | 30-39 | Both | Standard 2-Tier (first step) | ELISA IgG VlsE | 3.8 (2-7.1) |  |
|  |  |  |  |  |  |  |  |  | ELISA IgM VlsE | 6.4 (3.9-10.3) |  |
|  |  |  |  | 414 |  | 40-49 | Both | Standard 2-Tier (first step) | ELISA IgG VlsE | 8.0 (5.8-11) |  |
|  |  |  |  |  |  |  |  |  | ELISA IgM VlsE | 8.0 (5.8-11) |  |
|  |  |  |  | 344 |  | 50-59 | Both | Standard 2-Tier (first step) | ELISA IgG VlsE | 12.5 (9.4-16.4) |  |
|  |  |  |  |  |  |  |  |  | ELISA IgM VlsE | 8.4 (5.9-11.8) |  |
|  |  |  |  | 110 |  | 60-69 | Both | Standard 2-Tier (first step) | ELISA IgG VlsE | 23.6 (16.6-32.3) |  |
|  |  |  |  |  |  |  |  |  | ELISA IgM VlsE | 14.5 (9.1-22.3) |  |
| Norway | Vestrheim 2016 ^4^ | Cross-sectional – “concordance” study (2011-2013) | Not described | 3057 | Residual sera (pertussis study) | ≥2 | Both | Single Tier | ELISA IgG VlsE | 2.7 (2.2-3.3) |  |
|  |  |  |  |  |  |  |  | Single Tier | EIA IgG | 3.4 (2.8-4.1) |  |
|  |  |  |  | 412 |  | 2-4 | Both | Single Tier | ELISA IgG VlsE | 1.7 (0.8-3.5) |  |
|  |  |  |  |  |  |  |  | Single Tier | EIA IgG | 3.6 (2.2-5.9) |  |
|  |  |  |  | 655 |  | 5-9 | Both | Single Tier | ELISA IgG VlsE | 3.7 (2.5-5.4) |  |
|  |  |  |  |  |  |  |  | Single Tier | EIA IgG | 4.1 (2.8-5.9) |  |
|  |  |  |  | 1213 |  | 10-19 | Both | Single Tier | ELISA IgG VlsE | 1.5 (1-2.4) |  |
|  |  |  |  |  |  |  |  | Single Tier | EIA IgG | 2.1 (1.4-3.1) |  |
|  |  |  |  | 460 |  | 20-39 | Both | Single Tier | ELISA IgG VlsE | 3.5 (2.2-5.6) |  |
|  |  |  |  |  |  |  |  | Single Tier | EIA IgG | 3.7 (2.3-5.8) |  |
|  |  |  |  | 317 |  | ≥50 | Both |  | ELISA IgG VlsE | 6 (3.9-9.2) |  |
|  |  |  |  |  |  |  |  | Single Tier | EIA IgG | 6 (3.9-9.2) |  |
| **Southern Europe** | |  |  |  |  |  |  |  |  |  |  |
| Spain | Barreiro-Hurle 2020 ^5^ | Cross-sectional (2014) | Convenience | 316 | Blood donors | Mean (±SD): 46 (± 8.5) | Both | Standard 2-Tier | ELISA + WB IgG | 5.1 (3.2-8.1) |  |
|  |  |  |  | 131 |  |  | Female | Standard 2-Tier | ELISA + WB IgG | 3.1 (1.2-7.7) |  |
|  |  |  |  | 185 |  |  | Male | Standard 2-Tier | ELISA + WB IgG | 6.5 (3.8-11) |  |
|  |  |  |  | 432 | Patients without infectious disease | Mean (±SD): 54 (± 14.1) | Both | Standard 2-Tier | ELISA + WB IgG | 14.4 (11.4-18) |  |
|  |  |  |  | 222 |  |  | Female | Standard 2-Tier | ELISA + WB IgG | 9.5 (6.3-14.1) |  |
|  |  |  |  | 210 |  |  | Male | Standard 2-Tier | ELISA + WB IgG | 19.5 (14.7-25.4) |  |
| Turkey | Uyanik 2009 ^6^ | Prospective cohort (2007-2008) | Convenience | 101 | Residents of high-risk area (Erzurum Province) |  | Both | Modified 2-Tier ^d^ | ELISA + ELFA IgG | 2 (0.6-7) |  |
|  |  |  |  | 61 |  | Mean age: 39.5 | Male | Modified 2-Tier ^d^ | ELISA+ ELFA IgG | 6.1 (2.2-15.1) |  |
|  |  |  |  | 40 |  | Mean age: 33.7 | Female | Modified 2-Tier ^d^ | ELISA+ ELFA IgG | 2.5 (0.4-12.9) |  |
|  |  |  |  | 79 | Blood donors |  | Both | Modified 2-Tier ^d^ | ELISA + ELFA IgG | 2.5 (0.7-8.7) |  |
|  |  |  |  | 40 |  | Mean age: 37.9 | Male | Modified 2-Tier ^d^ | ELISA+ ELFA IgG | 2.6 (0.4-13) |  |
|  |  |  |  | 39 |  | Mean age: 35.8 | Female | Modified 2-Tier ^d^ | ELISA+ ELFA IgG | 2.5 (0.4-13.1) |  |
| Turkey | Akar N 2018 ^7^ | Cross-sectional (2016) | Convenience | 193 | Residents of a high-risk area (Duzce) | Mean (±SD): 47.4 (± 13.5) | Both | Standard 2-Tier | ELISA + WB IgM | 1.5 (0.5-4.4) |  |
|  |  |  |  |  |  |  |  | Standard 2-Tier | ELISA + WB IgG | 6.2 (3.6-10.5) |  |
|  |  |  |  | 103 |  |  | Female | Standard 2-Tier | ELISA + WB IgM | 1 (0.2-5.3) |  |
|  |  |  |  |  |  |  |  | Standard 2-Tier | ELISA + WB IgG | 9 (4.9-16.1) |  |
|  |  |  |  | 90 |  |  | Male | Standard 2-Tier | ELISA + WB IgM | 2 (0.5-7.4) |  |
|  |  |  |  |  |  |  |  | Standard 2-Tier | ELISA + WB IgG | 3 (1-8.9) |  |
|  |  |  |  | 83 |  | 18-45 | Both | Standard 2-Tier | ELISA + WB IgM | 0 (0-4.4) |  |
|  |  |  |  |  |  |  |  | Standard 2-Tier | ELISA + WB IgG | 2 (0.5-7.8) |  |
|  |  |  |  | 110 |  | 16-70 | Both | Standard 2-Tier | ELISA + WB IgM | 3 (1.1-8.1) |  |
|  |  |  |  |  |  |  |  | Standard 2-Tier | ELISA + WB IgG | 9 (5-15.8) |  |
| Turkey | Parlak 2015 ^8^ | Cross-sectional (2012) | Cluster (random sampling in clusters) | 446 | Residents of a high-risk area (Van region) | Mean (±SD): 39.6 (± 15.5) | Both | Standard 2-Tier | ELISA + WB IgG | 0.89 (0.4-2.3) |  |
|  |  |  |  | 307 |  | Mean (±SD): 39.1 (± 15.1) | Female | Standard 2-Tier | ELISA + WB IgG | 1.3 (0.5-3.3) |  |
|  |  |  |  | 139 |  | Mean (±SD): 40.9 (± 16.3) | Male | Standard 2-Tier | ELISA + WB IgG | 0 (0-2.7) |  |
| Turkey | Basbulut 2012 ^9^ | Cross-sectional (2006) | Cluster (random sampling in clusters) | 419 | Tekkeköy (high tick population) | Mean (±SD): 33.07 (± 19.58) | Both | Standard 2-Tier | ELISA + WB IgG | 3.3 (2-5.5) |  |
|  |  |  |  | 172 |  |  | Male | Standard 2-Tier | ELISA + WB IgG | 2.3 (0.9-5.8) |  |
|  |  |  |  | 247 |  |  | Female | Standard 2-Tier | ELISA + WB IgG | 4 (2.2-7.2) |  |
|  |  |  |  | 313 |  | ≤ 45 | Both | Standard 2-Tier | ELISA + WB IgG | 2.9 (1.5-5.4) |  |
|  |  |  |  | 106 |  | > 45 | Both | Standard 2-Tier | ELISA + WB IgG | 4.7 (2-10.6) |  |
| Turkey | Bucak 2016 ^10^ | Cross-sectional (Aug-Oct 2013) | Stratified random | 196 | Residents of Bolu | Adults and children | Both | Standard 2-Tier | ELISA + WB IgM | 3.5 (1.7-7.1) |  |
|  |  |  |  |  |  |  |  | Standard 2-Tier | ELISA + WB IgG | 4.5 (2.4-8.4) |  |
|  |  |  |  |  |  |  |  | Standard 2-Tier | ELISA + WB IgG or IgM | 8.1 (5-12.8) |  |
|  |  |  |  | 120 |  |  | Female | Standard 2-Tier | ELISA + WB IgG or IgM | 8.3 (4.6-14.6) |  |
|  |  |  |  | 76 |  |  | Male | Standard 2-Tier | ELISA + WB IgG or IgM | 7.9 (3.7-16.2) |  |
|  |  |  |  | 4 |  | 0-20 | Both | Standard 2-Tier | ELISA + WB IgG or IgM | 0 (0-49) |  |
|  |  |  |  | 62 |  | 21-40 | Both | Standard 2-Tier | ELISA + WB IgG or IgM | 6.5 (2.6-15.5) |  |
|  |  |  |  | 50 |  | 41-60 | Both | Standard 2-Tier | ELISA + WB IgG or IgM | 10 (4.4-21.4) |  |
|  |  |  |  | 80 |  | >60 | Both | Standard 2-Tier | ELISA + WB IgG or IgM | 8.8 (4.3-17) |  |
| Turkey | Cikman 2018 ^11^ | Cross-sectional (2014) | Cluster | 368 | Residents of Erzincan (high tick population) | Mean (±SD): 51.43 (±16.91) | Both | Standard 2-Tier | ELISA + WB IgG | 2.1 (1.1-4.1) |  |
|  |  |  |  | 225 |  |  | Female | Standard 2-Tier | ELISA + WB IgG | 2.2 (0.9-5.1) |  |
|  |  |  |  | 143 |  |  | Male | Standard 2-Tier | ELISA + WB IgG | 2 (0.7-5.8) |  |
| Turkey | Cora 2017 ^12^ | Retrospective, cross-sectional (2007-2008) | Convenience | 884 | Residents of Trabzon city | Range: 20-79 | Both | Standard 2-Tier | ELISA + WB IgG | 14.5 (12.3-17) |  |
|  |  |  |  | 555 | High risk occupation |  | Both | Standard 2-Tier | ELISA + WB IgG | 15.8 (13-19.1) |  |
|  |  |  |  | 329 | Low risk occupation |  | Both | Standard 2-Tier | ELISA + WB IgG | 12.2 (9.1-16.2) |  |
|  |  |  |  | 453 | Residents of Trabzon city |  | Female | Standard 2-Tier | ELISA + WB IgG | 15 (12-18.6) |  |
|  |  |  |  | 431 |  |  | Male | Standard 2-Tier | ELISA + WB IgG | 13.9 (11-17.5) |  |
|  |  |  |  | 262 | Residents of Trabzon city | 20-29 | Both | Standard 2-Tier | ELISA + WB IgG | 8.8 (5.9-12.9) |  |
|  |  |  |  | 203 |  | 30-39 | Both | Standard 2-Tier | ELISA + WB IgG | 10.3 (6.8-15.2) |  |
|  |  |  |  | 173 |  | 40-49 | Both | Standard 2-Tier | ELISA + WB IgG | 15 (10.4-21.1) |  |
|  |  |  |  | 122 |  | 50-59 | Both | Standard 2-Tier | ELISA + WB IgG | 19.7 (13.6-27.6) |  |
|  |  |  |  | 69 |  | 60-69 | Both | Standard 2-Tier | ELISA + WB IgG | 23.2 (14.8-34.4) |  |
|  |  |  |  | 55 |  | >70 | Both | Standard 2-Tier | ELISA + WB IgG | 32.7 (21.8-45.9) |  |
| **United Kingdom** | | | | | | |  |  |  |  |  |
| Scotland | Munro 2015 ^13^ | Cross-sectional (2010-2011) | Convenience | 734 | Blood donors |  | Males | Standard 2-Tier | ELISA IgG and/or IgM +WB IgG | 4.0 (2.9-5.7) |  |
|  |  |  |  | 706 |  |  | Females | Standard 2-Tier | ELISA IgG and/or IgM +WB IgG | 4.4 (3.2-6.2) |  |
| **Western and Central Europe** | | | |  |  |  |  |  |  |  |  |
| Austria | Cetin 2006 ^14^ | Cross-sectional (2002-2003) | Convenience | 1253 | Hunters | Mean (±SD): 51 (±13) | Both | Standard 2-Tier | ELISA + WB IgG | 53.7 (51-56.5) |  |
|  |  |  |  | 79 |  | <29 | Both | Standard 2-Tier | ELISA+ WB IgG | 33 (23.6-43.9) |  |
|  |  |  |  | 194 |  | 30-39 | Both | Standard 2-Tier | ELISA+ WB IgG | 29.8 (23.8-36.6) |  |
|  |  |  |  | 335 |  | 40-49 | Both | Standard 2-Tier | ELISA+ WB IgG | 45.3 (40.1-50.7) |  |
|  |  |  |  | 273 |  | 50-59 | Both | Standard 2-Tier | ELISA+ WB IgG | 59.3 (53.4-65) |  |
|  |  |  |  | 300 |  | 60-69 | Both | Standard 2-Tier | ELISA+ WB IgG | 71.6 (66.3-76.4) |  |
|  |  |  |  | 72 |  | >70 | Both | Standard 2-Tier | ELISA+ WB IgG | 83.3 (73.1-90.2) |  |
| Belgium | Keukeleire 2016 ^15^ | Cross-sectional (2011) | Convenience | 127 | Veterinarians and farmers | ≥ 25 | Both | Single Tier | ELISA IgG | 5.5 (2.7-10.9) |  |
|  |  |  |  | 31 | Farmers |  | Both | Single Tier | ELISA IgG | 9.68 (3.4-24.9) |  |
|  |  |  |  | 96 | Veterinarians |  | Both | Single Tier | ELISA IgG | 4.17 (1.6-10.2) |  |
|  |  |  |  | 109 | Veterinarians and farmers* |  | Male | Single Tier | ELISA IgG | 6.4 (3.1-12.6) |  |
|  |  |  |  | 18 |  |  | Female | Single Tier | ELISA IgG | 0 (0-17.6) |  |
|  |  |  |  | 31 |  | 25-39 | Both | Single Tier | ELISA IgG | 0 (0-11) |  |
|  |  |  |  | 30 |  | 40-49 | Both | Single Tier | ELISA IgG | 3.33 (0.6-16.7) |  |
|  |  |  |  | 45 |  | 50-59 | Both | Single Tier | ELISA IgG | 8.8 (3.5-20.6) |  |
|  |  |  |  | 20 |  | 60-72 | Both | Single Tier | ELISA IgG | 10 (2.8-30.1) |  |
| Belgium | Lernout 2019 ^16^ | Cross-sectional (2013-2015) | Convenience | 1608 | Serum bank samples |  | Male | Standard 2-Tier | CLIA IgG + WB IgG | 1 .4 (0.9-2.1) |  |
|  |  |  |  | 1607 |  |  | Female | Standard 2-Tier | CLIA IgG + WB IgG | 0.8 (0.5-1.4) |  |
|  |  |  |  | 1271 |  | < 15 |  | Standard 2-Tier | CLIA IgG + WB IgG | 0.9 (0.5-1.6) |  |
|  |  |  |  | 1590 |  | 15–59 |  | Standard 2-Tier | CLIA IgG + WB IgG | 1.1 (0.7-1.7) |  |
|  |  |  |  | 354 |  | ≥ 60 |  | Standard 2-Tier | CLIA IgG + WB IgG | 1.9 (1.0-4.0) |  |
| France | Thorin 2008 ^17^ | Cross-sectional (2002-2003) | Convenience | 2975 | Forest field professionals | Range: 17-81 | Both | Standard 2-Tier | ELISA + WB IgG and/or IgM | 14.1 (12.9-15.4) |  |
|  |  |  |  | 2916 |  |  | Male | Standard 2-Tier | ELISA+WB IgG and/or IgM | 14.3 (13.1-15.6) |  |
|  |  |  |  | 59 |  |  | female | Standard 2-Tier | ELISA+WB IgG and/or IgM | 3.4 (0.9-11.6) |  |
|  |  |  |  | 48 |  | <20 | Both | Standard 2-Tier | ELISA+WB IgG and/or IgM | 4.2 (1.2-14) |  |
|  |  |  |  | 205 |  | 20-24 | Both | Standard 2-Tier | ELISA+WB IgG and/or IgM | 2.4 (1-5.5) |  |
|  |  |  |  | 226 |  | 25-29 | Both | Standard 2-Tier | ELISA+WB IgG and/or IgM | 1.8 (0.7-4.5) |  |
|  |  |  |  | 282 |  | 30-34 | Both | Standard 2-Tier | ELISA+WB IgG and/or IgM | 7.1 (4.6-10.7) |  |
|  |  |  |  | 383 |  | 35-49 | Both | Standard 2-Tier | ELISA+WB IgG and/or IgM | 9.7 (7.1-13.1) |  |
|  |  |  |  | 535 |  | 40-44 | Both | Standard 2-Tier | ELISA+WB IgG and/or IgM | 14.4 (11.7-17.6) |  |
|  |  |  |  | 537 |  | 45-49 | Both | Standard 2-Tier | ELISA+WB IgG and/or IgM | 19.9 (16.7-23.5) |  |
|  |  |  |  | 424 |  | 50-54 | Both | Standard 2-Tier | ELISA+WB IgG and/or IgM | 19.3 (15.8-23.3) |  |
|  |  |  |  | 278 |  | 55-59 | Both | Standard 2-Tier | ELISA+WB IgG and/or IgM | 23.7 (19.1-29) |  |
|  |  |  |  | 42 |  | 60-64 | Both | Standard 2-Tier | ELISA+WB IgG and/or IgM | 33.3 (21-48.4) |  |
|  |  |  |  | 18 |  | ≥65 | Both | Standard 2-Tier | ELISA+WB IgG and/or IgM | 27.8 (12.5-50.9) |  |
| France | Ruiz 2020 ^18^ | Cohort study  (2007-2016) | Random | 432 | Retired farmers | 65 years or older | Males | Standard 2-Tier | ELISA IgG + WB IgG | 9 (6.6-12.1) |  |
|  |  |  |  | 257 |  |  | Females | Standard 2-Tier | ELISA IgG + WB IgG | 2.3 (1.0-5.0) |  |
| Germany | Wilking 2015 ^19^ | Cross-sectional (2008-2011) | Not described- survey assessed the health status | 6945 | Global population | ≥ 18 | Both | Standard 2-Tier | ELISA + WB IgG | 10.6 (9.9-11.4) |  |
|  |  |  |  | 3331  3614 |  |  | Male  Female | Standard 2-Tier | ELISA +WB IgG  ELISA + WB IgG | 15 (13.8-16.3)  6.6 (5.8-7.5) |  |
|  |  |  |  | 1043 |  | 18-29 | Both | Standard 2-Tier | ELISA + WB IgG | 5.9 (4.6-7.5) |  |
|  |  |  |  | 829 |  | 30-39 | Both | Standard 2-Tier | ELISA + WB IgG | 6 (4.6-7.8) |  |
|  |  |  |  | 1263 |  | 40-49 | Both | Standard 2-Tier | ELISA + WB IgG | 6.5 (5.3-8) |  |
|  |  |  |  | 1373 |  | 50-59 | Both | Standard 2-Tier | ELISA + WB IgG | 9.1 (7.7-10.7) |  |
|  |  |  |  | 1361 |  | 60-69 | Both | Standard 2-Tier | ELISA + WB IgG | 13.6 (11.9-15.5) |  |
|  |  |  |  | 1067 |  | 70-79 | Both | Standard 2-Tier | ELISA + WB IgG | 21.9 (19.5-24.5) |  |
| Poland | Zajac 2017 ^20^ | Cross-sectional (2015-2016) | Convenience | 3597 | Farmers | Mean (±SD): 51.3  (± 11.4) | Both | Single Tier | ELISA IgM | 11.5 (10.5-12.6) |  |
|  |  |  |  |  |  |  |  |  | ELISA IgG | 13.7 (12.6-14.9) |  |
|  |  |  |  | 2128 |  |  | Female | Single Tier | ELISA IgM | 12.1 (10.8-13.6) |  |
|  |  |  |  |  |  |  |  |  | ELISA IgG | 11.7 (10.4-13.1) |  |
|  |  |  |  | 1469 |  |  | Male | Single Tier | ELISA IgM | 10.6 (9.1-12.3) |  |
|  |  |  |  | 1469 |  |  |  |  | ELISA IgG | 16.5 (14.7-18.5) |  |
|  |  |  |  | 127 |  | 18-29 | Both | Single Tier | ELISA IgM | 13.4 (8.5-20.4) |  |
|  |  |  |  | 127 |  |  |  |  | ELISA IgG | 6.3 (3.2-11.5) |  |
|  |  |  |  | 440 |  | 30-39 | Both | Single Tier | ELISA IgM | 14.5 (11.5-18.1) |  |
|  |  |  |  | 440 |  |  |  |  | ELISA IgG | 6.8 (4.8-9.6) |  |
|  |  |  |  | 1001 |  | 40-49 | Both | Single Tier | ELISA IgM | 12.5 (10.6-14.7) |  |
|  |  |  |  | 1001 |  |  |  |  | ELISA IgG | 10.8 (9-12.9) |  |
|  |  |  |  | 1268 |  | 50-59 | Both | Single Tier | ELISA IgM | 10.3 (8.8-12.1) |  |
|  |  |  |  | 1268 |  |  |  |  | ELISA IgG | 14.2 (12.4-16.2) |  |
|  |  |  |  | 577 |  | 60-69 | Both | Single Tier | ELISA IgM | 10.4 (8.2-13.2) |  |
|  |  |  |  | 577 |  |  |  |  | ELISA IgG | 19.2 (16.2-22.6) |  |
|  |  |  |  | 156 |  | 70-79 | Both | Single Tier | ELISA IgM | 7.7 (4.5-13) |  |
|  |  |  |  | 156 |  |  |  |  | ELISA IgG | 28.2 (21.7-35.7) |  |
|  |  |  |  | 28 |  | 80-91 | Both | Single Tier | ELISA IgM | 14.3 (5.7-31.5) |  |
|  |  |  |  | 28 |  |  |  |  | ELISA IgG | 35.7 (20.7-54.2) |  |

Seroprevalence estimates and 95% CIs have been rounded to the first decimal to the right for consistent level of precision C6, C6 protein of the variable major-protein-like sequence lipoprotein; EIA, enzyme immunoassay; IFA, immunofluorescence assay; ELISA, enzyme-linked immunosorbent assay; SP, seropositivity; WB, Western Blot; * Results reported from the study according to age groups of veterinarians and farmers n = 127, however the study within the age group only includes 126 participants

*Footnotes:*

^a^ Includes single test results and two-tier testing overall results based on standard or modified algorithm ^21,22^

^b^ Cluster sampling: methodology that involves: 1) dividing the population into subgroups or clusters that are not necessarily (and preferably not) homogeneous; 2) drawing a random sample of the clusters; and 3) selecting all or a random sample of the persons in each cluster

^c^ Sample taken ~50 years ago and then tested with current diagnostic test

^d^ Modified based on current guidelines for those articles published after modified testing strategies were published ^21^.

**Table S2. Comparison of Seroprevalence of Lyme Borreliosis estimates among high and low risk groups in Europe, with measures of effect (Odds Ratios)**

| **Country** | **Reference** | **Sample size (N1/N2)^a^** | **Type of diagnostic test** | **High risk** | **Final SP result ^b^** | **Low risk** | **Final SP result ^b^** | **OR (95% CI)** |
| --- | --- | --- | --- | --- | --- | --- | --- | --- |
| Slovenia | Rojko 2005 ^23^ | 112/93 | ELISA IgG | Forestry workers | 25.8 | Indoor workers from the same region | 9.7 | 3.2 (1.5-7.4)* |
|  |  |  | ELISA IgM |  | 16.4 |  | 16.2 | 1.0 (0.5-2.1) |
|  |  |  | IFA |  | 9.8 |  | 4.3 | 2.4 (0.8-8.3) |
| Italy | Tomao 2005 ^24^ | 412/365 | ELISA+WB IgG and/or IgM ^c^ | Forest workers | 7 | Blood donors | 3.5 | 2.1 (1.1-4.1)* |
|  |  |  | ELISA+WB IgG and/or IgM ^`1^ |  | 3.8 |  | 1.6 | 2.4 (1-6.6) |
| Italy | Di Renzi 2010 ^25^ | 145/282 | ELISA+WB IgG | Forestry rangers | 0.68 | Blood donors | 1.06 | 0.7 (0.1-4.9) |
|  |  |  | ELISA+WB IgM |  | 13.1 |  | 8.16 | 1.7 (0.9-3.2) |
| Serbia | Jovanovic 2015 ^26^ | 34/35 | ELISA+WB IgM and/or IgG | Forestry workers | 11.76 | Blood donors | 8.57 | 1.4 (0.3-7.1) |
| Serbia | Krstic 2007 ^27^ | 34/35 | ELISA IgM and/or IgG | Professionally exposed (public utility workers) | 23.5 | Not professionally exposed (military medical cadets) | 2.9 | 8.9 (1.6-102.7)* |
| Turkey | Uyanik 2009 ^6^ | 101/79 | ELISA+ELFA IgG | Residents of high-risk area (Erzurum Province) | 2.0 | Blood donors | 2.5 | 0.8 (0.1-5.7) |
| Turkey | Gunes 2005 ^28^ | 270/135 | ELISA IgG | Persons with contact with livestock | 0.4 | Healthy controls | 0.7 | 0.6 (0.0-8.5) |
| Turkey | Cora 2017 ^12^ | 555/329 | ELISA+WB IgG | High risk occupation | 15.8 | Low risk occupation | 12.2 | 1.3 (1.0-2.0) |
| Poland | Tokarska-Rodak 2014 ^29^ | 172/45 | ELISA+WB IgG and/or IgM | Forestry workers | 54.9 | Persons not professionally exposed | 5.0 | 21.5 (6.9-105.5)* |
|  |  | 104/45 |  | Farmers living in the region | 28 | Persons not professionally exposed | 5.0 | 6.9 (2.1-34.4)* |
| Poland | Cisak 2008 ^30^ | 94/50 | ELISA+WB IgG, IgM | Farmers | 32.9 | Blood donors | 6.0 | 7.3 (2.5-29.4)* |
| Poland | Buczek 2009 ^31^ | 864/291 | ELISA IgM | Forestry workers | 13.8 | Office workers | 10.0 | 1.4 (0.9-2.2) |
|  |  |  | ELISA IgG |  | 25.0 |  | 13.7 | 2.1 (1.5-3.0)* |
| Slovakia | Zakutna 2015 B ^32^ | 193/36 | ELISA IgG | Agriculture and forestry workers | 29.2 | Police and border customs agents | 11.1 | 3.2 (1.2-10.6)* |
|  |  | 48/36 |  | Persons frequently staying in the countryside | 20.8 | Police and border customs agents | 11.1 | 2.1 (0.6-7.7) |

Seroprevalence estimates and 95% CIs have been rounded to the first decimal to the right for consistent level of precision SP, seroprevalence; WB: Western Blot; OR: Odds Ratio; CI: Confidence Intervals; * indicates OR is statistically significant

^a^ Number of persons tested in the high-risk **(N1)**/low-risk groups **(N2);** ^b^ Includes single test results and 2-tier testing overall results based on standard or modified algorithm ^21,22^; ^c^ According to manufacturer’s instructions; ^d^ According to CDC-recommended criteria; ^e^ studies comparing populations in similar risk categories

**Table S3. Distribution of articles by risk category and study design**

| **Type of risk** | **Study design** | **n (%)** | **Author Year (Publications)** |
| --- | --- | --- | --- |
| High-risk groups (n=34) | **Cross-sectional** | 27 (79.4) | Parm 2015, Motiejunas 2009, Lakos 2012, Krstic 2007, Lledo 2019, Akar N 2018, Parlak 2015, Basbulut 2012, Cikman 2018, Gunes 2005, Cora 2017, Cetin 2006, Keukeleire 2016, De Keukeleire 2018, Thorin 2008, Tokarska-Rodak 2014, Kocbach 2014, Bura 2018, Cisak 2008, Zajac 2017, Buczek 2009, Kiewra 2018, Zakutna 2015 B, Rojko 2005, Busova 2018, Pańczuk 2019, Gazi 2016 |
|  | **Cohort** | 7 (20.5) | Kaya 2008, Tomao 2005, Di Renzi 2010, Jovanovic 2015, Uyanik 2009, Podsiadly 2011, Ruiz 2020 |
| General population (n=39) | **Cross-sectional** | 33 (84.6) | Magnaval 2016, Biletska 2008, Cuellar 2019, Van Beek 2017, Hjetland 2014, Vestrheim 2016, Carlsson 2018, Krstic 2007, Barreiro-Hurle 2020, Oteiza-Olaso 2011, Bucak 2016, Gunes 2005, Cora 2017, Sonnleitner 2015, Kriz 2018 A, Hajek 2006, Wilking 2015, Dehnert 2012, Van Gorkom 2017, Tokarska-Rodak 2014, Machcinska 2013, Pawelczyk 2019, Cisak 2008, Buczek 2009, Zakutna 2015 A, Bazovska 2010, Rojko 2005, Munro 2015, Lernout 2019, Kuchynka 2016, Bazovska 2005, Thortveit 2020, Johansson 2017 |
|  | **Cohort** | 6 (15.3) | Kaya 2008, Tomao 2005, Di Renzi 2010, Jovanovic 2015, Uyanik 2009, Bozkurt 2008 |

Seroprevalence estimates and 95% CIs have been rounded to the first decimal to the right for consistent level of precision

*Total N>61, as some studies contain both groups.

**Table S4. Number of publications by countries in European regions** ^33^

| **European region** | **Countries** | **Number of publications** |
| --- | --- | --- |
| **Eastern Europe** | Czech Republic, Hungary, Romania, Russian Federation, Slovakia, Slovenia, Poland, Ukraine | 23 |
| **Northern Europe** | |  |
| ***Baltic states*** | Estonia, Lithuania | 2 |
| ***Scandinavia*** | Denmark, Finland, Norway, Sweden | 7 |
| ***The United Kingdom & Ireland*** | England, Northern Ireland, Scotland, Republic of Ireland, Wales | 1 |
| **Southern Europe** | Italy, Portugal, Serbia, Turkey | 18 |
| **Western Europe** | Austria, Belgium, France, Germany, the Netherlands | 10 |
| Seroprevalence estimates and 95% CIs have been rounded to the first decimal to the right for consistent level of precision |  |  |

**Table S5. Criteria used to classify participants in studies by their risk of exposure to ticks**

| **Level of tick exposure** | **Criteria** |
| --- | --- |
| **High** | Hunters  Forestry workers  Field workers  Veterinarians  Farmers  Retired farmers  Soldiers  People with occupational activities in nature  Residents of areas endemic for LB |
| **Low or unknown** | Office workers  Employed in indoor job  Not occupationally exposed to ticks  Occupation unrelated to livestock and rural areas  Healthy adult volunteers  Blood donors  Residual sera from other studies  No history of previous LB  Blood donors with a process not related to cause Infectious disease  Patients with other diseases (HIV-infected, psychiatric, Adults with normal left ventricular systolic function and no history suggestive of myocarditis) |

Seroprevalence estimates and 95% CIs have been rounded to the first decimal to the right for consistent level of precision

**References**

1. Parm Ü, Niitvägi E, Beljaev K, et al. Lyme borreliosis in Saaremaa. Eesti Arst 2015;94(4):203-210.

2. Zhang R, Gong T, Chen T, et al. Re: 'Seroprevalence of Lyme borreliosis in Finland 50 years ago' by Cuellar et al. Clin Microbiol Infect 2020;26(7):949-950.

3. Hjetland R, Nilsen RM, Grude N, et al. Seroprevalence of antibodies to *Borrelia burgdorferi sensu lato* in healthy adults from western Norway: risk factors and methodological aspects. APMIS 2014;122(11):1114-1124.

4. Vestrheim DF, White RA, Aaberge IS, et al. Geographical differences in seroprevalence of *Borrelia burgdorferi* antibodies in Norway, 2011-2013. Ticks Tick Borne Dis 2016;7(5):698-702.

5. Barreiro-Hurle L, Melon-Garcia S, Seco-Bernal C, et al. Seroprevalence of Lyme disease in southwest Asturias. Enferm Infecc Microbiol Clin 2020;38(4):155-158.

6. Uyanık MH, Yazgı H, Ayyıldız A. Seropositivity of Lyme disease in Erzurum Province, Turkey. İnfeksiyon Dergisi = Turkish Journal of Infection 2009;23(2):69-72.

7. Akar N, Çalişkan E, Öztürk CE, et al. Seroprevalence of hantavirus and borrelia burgdorferi in düzce (Turkey) forest villages and the relationship with sociodemographic features. Turkish Journal of Medical Sciences 2019;49(2):483-489.

8. Parlak M, Bayram Y, Çıkman A, et al. [Seropositivity of *Borrelia burgdorferi* in risky groups in Van region, Turkey]. Mikrobiyol Bul 2015;49(3):439-445.

9. Aslan Başbulut E, Gözalan A, Sönmez C, et al. Seroprevalence of Borrelia burgdorferi and tick-borne encephalitis virus in a rural area of Samsun, Turkey. Mikrobiyoloji Bulteni 2012;46(2):247-256.

10. Bucak Ö, Koçoğlu ME, Taș T, et al. Evaluation of Borrelia burgdorferi sensu lato seroprevalence in the province of Bolu, Turkey. Turkish Journal of Medical Sciences 2016;46(3):727-732.

11. Cikman A, Aydin M, Gulhan B, et al. Geographical Features and Seroprevalence of Borrelia burgdorferi in Erzincan, Turkey. Journal of Arthropod-Borne Diseases 2019.

12. Cora M, Kaklıkkaya N, Topbaș M, et al. Determination of seroprevalence of Borrelia burgdorferi IgG in adult population living in Trabzon. Balkan Medical Journal 2017;34(1):47-52.

13. Munro H, Mavin S, Duffy K, et al. Seroprevalence of lyme borreliosis in Scottish blood donors. (1365-3148 (Electronic)).

14. Cetin E, Sotoudeh M, Auer H, et al. Paradigm Burgenland: risk of *Borrelia burgdorferi sensu lato* infection indicated by variable seroprevalence rates in hunters. Wien Klin Wochenschr 2006;118(21-22):677-681.

15. Keukeleire Md, Robert A, Kabamba B, et al. Individual and environmental factors associated with the seroprevalence of Borrelia burgdorferi in Belgian farmers and veterinarians. Infection Ecology &amp; Epidemiology 2016;6:32793.

16. Lernout TA-O, Kabamba-Mukadi B, Saegeman V, et al. The value of seroprevalence data as surveillance tool for Lyme borreliosis in the general population: the experience of Belgium. (1471-2458 (Electronic)).

17. Thorin C, Rigaud E, Capek I, et al. Seroprevalence of Lyme borreliosis and tick-borne encephalitis in workers at risk, in Eastern France. Médecine et Maladies Infectieuses 2008;38(10):533-542.

18. Ruiz VH, Edjolo A, Roubaud-Baudron C, et al. Association of Seropositivity to Borrelia burgdorferi With the Risk of Neuropsychiatric Disorders and Functional Decline in Older Adults: The Aging Multidisciplinary Investigation Study. (2168-6157 (Electronic)).

19. Wilking H, Fingerle V, Klier C, et al. Antibodies against Borrelia burgdorferi sensu lato among Adults, Germany, 2008–2011. Emerging Infectious Diseases 2015;21(1):107-110.

20. Zając V, Pinkas J, Wójcik-Fatla A, et al. Prevalence of serological response to Borrelia burgdorferi in farmers from eastern and central Poland. European Journal of Clinical Microbiology &amp; Infectious Diseases 2017;36(3):437-446.

21. Branda JA, Steere AC. Laboratory Diagnosis of Lyme Borreliosis. Clin Microbiol Rev 2021;34(2).

22. Marques AR, Strle F, Wormser GP. Comparison of Lyme disease in the United States and Europe. Emerg Infect Dis 2021;27(8):2017-2024.

23. Rojko T, Ruzic-Sabljic E, Strle F, et al. Prevalence and incidence of Lyme borreliosis among Slovene forestry workers during the period of tick activity. Wien Klin Wochenschr 2005;117(5-6):219-225.

24. Tomao P, Ciceroni L, D'Ovidio MC, et al. Prevalence and incidence of antibodies to *Borrelia burgdorferi* and to tick-borne encephalitis virus in agricultural and forestry workers from Tuscany, Italy. Eur J Clin Microbiol Infect Dis 2005;24(7):457-463.

25. Di Renzi S, Martini A, Binazzi A, et al. Risk of acquiring tick-borne infections in forestry workers from Lazio, Italy. Eur J Clin Microbiol Infect Dis 2010;29(12):1579-1581.

26. Jovanovic D, Atanasievska S, Protic-Djokic V, et al. Seroprevalence of Borrelia burgdorferi in occupationally exposed persons in the Belgrade area, Serbia. Brazilian Journal of Microbiology 2015;46(3):807-814.

27. Krstić M, Stajković N. Risk for infection by lyme disease cause in green surfaces maintenance workers in Belgrade. Vojnosanitetski pregled. Military-medical and pharmaceutical review 2007;64(5):313-318.

28. Güneş T, Poyraz O, Kaya S, et al. Investigation of vectors for Borrelia burgdorferi and Lyme seropositivity in Sivas region. Mikrobiyoloji bülteni 2005;39(4):503-508.

29. Tokarska-Rodak M, Plewik D, Kozioł-Montewka M, et al. Risk of occupational infections caused by Borrelia burgdorferi among forestry workers and farmers. Medycyna Pracy 2014;65(1):109-118.

30. Cisak E, Chmielewska-Badora J, Zwoliñski J, et al. Study on Lyme borreliosis focus in the Lublin Region (Eastern Poland). Annals of Agricultural and Environmental Medicine 2008;15(2):327-332.

31. Buczek A, Rudek A, Bartosik K, et al. Seroepidemiological study of Lyme borreliosis among forestry workers in southern Poland. Annals of Agricultural and Environmental Medicine 2009;16(2):257-261.

32. Zákutná L, Dorko E, Mattová E, et al. Sero-epidemiological study of Lyme disease among high-risk population groups in eastern Slovakia. Annals of Agricultural and Environmental Medicine 2015;22(4):632-636.

33. World Health Organization. Annex: Regional Classifications. Available from: <https://cdn.who.int/media/docs/default-source/air-pollution-documents/air-quality-and-health/country-groupings-database-2022.pdf>. [Last accessed January 18, 2023].
